# Oral administration of *Lactiplantibacillus plantarum* displaying multiple ASFV antigen proteins on the surface induces systemic immune responses in mice

**DOI:** 10.1128/aem.00279-26

**Published:** 2026-04-24

**Authors:** Shuhui Fan, Mingyang Cheng, Lijia Wen, Anqi Hu, Boshi Zou, Tianming Niu, Mingxiao Liu, Yuxin Jiang, Yu Sun, Meiying Bao, Nan Wang, Hongliang Chen, Chunwei Shi, Chunfeng Wang

**Affiliations:** 1 College of Veterinary Medicine, Jilin Agricultural University 85112, Changchun, China; 2 Jilin Provincial Engineering Research Center of Animal Probiotics, Jilin Agricultural University 85112, Changchun, China; 3 Key Laboratory of Animal Production and Product Quality Safety of Ministry of Education, Jilin Agricultural University 85112, Changchun, China; 4 Jilin Provincial Key Laboratory of Animal Microecology and Healthy Breeding, Jilin Agricultural University 85112, Changchun, China; Centers for Disease Control and Prevention, Atlanta, Georgia, USA

**Keywords:** African swine fever, antibiotic-free selection system, mucosal immune responses

## Abstract

African swine fever (ASF) is a highly fatal viral disease that poses a severe threat to the global swine industry. Developing a safe and effective vaccine remains an urgent and complex challenge. Lactobacillus plantarum (*L. plantarum*), recognized as a food-grade antigen delivery vehicle, has demonstrated great promise in vaccine development and application for various infectious diseases. To overcome the weak intestinal immunogenicity and short mucosal residence of conventional vaccines, we employed the antibiotic-free selection system of *L. plantarum* NC8. By fusing the nontoxic, intestinal-epithelium–targeting cholera toxin B subunit (CTB) with key ASFV antigens (p30, p54, p49, p22, pK205R, and pE248R), we constructed five newly engineered lactic acid bacteria (LAB) strains which were subsequently administered to mice orally, either individually or as a multivalent cocktail. Experimental findings revealed that oral administration of the newly engineered LAB vaccine significantly promoted the activation of DCs within the intestinal mucosa. Moreover, this oral vaccine effectively triggered T-cell immune responses in both the mucosal and systemic immune compartments and markedly enhanced B-cell activation in the intestinal mucosa. Further immunological analyses confirmed that the vaccine significantly elevated serum antigen-specific IgG and intestinal antigen-specific IgA levels. Collectively, these results indicate that the cocktail of newly engineered LAB strains elicits potent humoral, cellular, and mucosal immune responses against ASFV in mice. This novel oral immunization strategy not only provides a strong theoretical foundation for developing an oral ASFV vaccine but also contributes valuable insights for advancing oral vaccines capable of inducing systemic immune protection through mucosal immunity.

**IMPORTANCE:**

African swine fever virus (ASFV) is a highly virulent pathogen that continues to inflict severe economic losses on the global swine industry. In this study, we developed an environmentally friendly oral vaccine candidate utilizing an antibiotic-free selection system based on *L. plantarum*. A cocktail of newly engineered LAB strains, each expressing distinct ASFV antigenic proteins (p30, p54, p49, p22, pK205R, and pE248R), was administered to mice via oral gavage to evaluate its protective potential against ASFV infection. The oral delivery of this newly engineered LAB cocktail elicited robust humoral, cellular, and mucosal immune responses, demonstrating significant enhancement of the host’s immunological defense mechanisms against ASFV. Overall, this strategy presents a promising approach for the prevention of ASFV infection while effectively eliminating the risks associated with antibiotic residues, thereby offering a safer and more sustainable vaccination platform for the swine industry.

## INTRODUCTION

African swine fever (ASF) is a highly fatal infectious disease recognized by the World Health Organization as a notifiable condition and poses a severe threat to the global swine industry (1). African swine fever virus (ASFV) possesses a large and complex genome, and the functions of many of its genes remain poorly understood, which has significantly impeded progress in vaccine development (2). Recent research has elucidated the structural features and assembly mechanisms of ASFV, providing crucial insights that facilitate rational vaccine design (3). Despite these advances, ASF continues to cause widespread economic losses worldwide, and the limited efficacy of existing vaccines and therapeutics underscores the urgent need to develop a safe and effective vaccine against this devastating disease.

Several studies have demonstrated that both humoral and cellular immune responses are essential for providing effective protection against ASFV infection (4). Consequently, current vaccine development strategies primarily focus on eliciting these two immune mechanisms simultaneously to achieve comprehensive and durable immunity. However, existing vaccines continue to exhibit notable limitations. For instance, although live-attenuated and viral vector-based vaccines can induce strong immune responses, they raise significant safety concerns, including the potential for chronic infections and adverse reactions (5). Similarly, while subunit vaccines are capable of generating neutralizing antibodies, such humoral responses alone are insufficient to confer complete protection (6). Therefore, it remains imperative to explore innovative vaccine platforms that can overcome these limitations and enable the development of safer, more effective ASFV vaccines.

In the pursuit of novel vaccine platforms, several key ASFV antigenic proteins have garnered increasing scientific attention. Research has demonstrated that recombinant p30, p72, and p54 proteins are capable of inducing the production of neutralizing antibodies in swine (6). Moreover, the proteins p49, p22, pK205R, and pE248R have shown strong potential as promising antigenic targets for preventing ASFV infection. For instance, studies have revealed that a recombinant vaccine expressing the p49 protein can partially inhibit ASFV infection, and viruses lacking p49 fail to maintain a complete icosahedral structure (7). Similarly, the p22 protein is closely associated with viral replication and pathogenicity, and immunization of pigs with recombinant baculovirus-expressed p22 has been shown to elicit neutralizing antibody responses (8). The pK205R protein also exhibits strong immunogenicity and reactivity; notably, it has been identified among 12 highly immunogenic antigens, including K205R, detected in the serum of convalescent pigs, highlighting its crucial role in ASFV prevention (9). The pE248R protein, encoded by the E248R gene, is equally important, as the absence of this protein in ASFV has been reported to reduce the virus’s infectivity and replication capacity by approximately 100-fold (10). Furthermore, studies have shown that a multivalent immunization approach, using a combination of type 2 adenoviruses expressing several antigens (p30, p54, CD2v, p72, and the p72 chaperone), delivered via intramuscular (IM) and intranasal (IN) routes, can protect pigs against lethal challenge with a virulent ASFV genotype I strain (11). Collectively, these findings suggest that a multi-antigen immunization strategy represents a promising and feasible approach for achieving effective protection against ASFV infection. However, there is currently no oral immunization strategy expressing multiple antigens.

ASFV can enter the host through mucosal surfaces; therefore, the establishment of effective mucosal immunity is crucial for preventing ASFV infection. In this context, the CTB has gained considerable attention as an ideal mucosal adjuvant due to its distinctive biological properties. CTB represents the non-toxic subunit of CT and forms a pentameric structure with strong binding affinity for monosialoganglioside GM1, which is abundantly expressed on intestinal epithelial cells (12). This property enables CTB to enhance the interaction between antigenic proteins and the mucosal surface when used as an adjuvant, thereby facilitating antigen delivery and promoting immune activation. Because of these unique characteristics, CTB has been extensively utilized in the design and development of vaccines targeting various viral and bacterial pathogens (13–16).

Mucosal immunity serves as the body’s first line of defense against respiratory and gastrointestinal viral infections (17). In this study, we demonstrated that fusing the mucosal adjuvant CTB with ASFV antigenic fusion proteins can elicit an enhanced mucosal immune response in mice. Our primary objective was to develop a *L. plantarum*-based vaccine targeting intestinal epithelial cells. To achieve this, six highly immunogenic ASFV antigens, p30, p54, p49, p22, pE248R, and pK205R, were selected as candidate antigens. Each ASFV antigen was fused with the CTB adjuvant and subsequently linked to the pSIP409-poly-γ-glutamic acid synthase A (pgsA′) plasmid, which contains the truncated pgsA′ gene from *Bacillus subtilis* known for its surface-anchoring properties. The resulting fusion protein was successfully displayed on the surface of *L. plantarum* NC8. Oral immunization with a mixture of these newly engineered LAB strains induced strong cellular and humoral immune responses in mice. Therefore, the oral administration of this newly engineered LAB mixture may provide effective theoretical protection against ASFV infection and offer valuable guidance for developing a new generation of ASF vaccines.

## RESULTS

### Construction of newly engineered LAB displaying ASFV antigen proteins on the surface

A critical consideration in probiotic development is the avoidance of antibiotic resistance genes to comply with safety standards for medical and food applications. To address this, we employed a complementary alr plasmid system to express ASFV antigen proteins in an alr-deficient *L. plantarum* NC8 strain (NC8Δ) (18). Due to the alanine racemase deficiency, this Δalr strain cannot sustain proliferation in environments lacking exogenous D-alanine supplementation, thereby making it more environmentally friendly.

**Fig 1:**
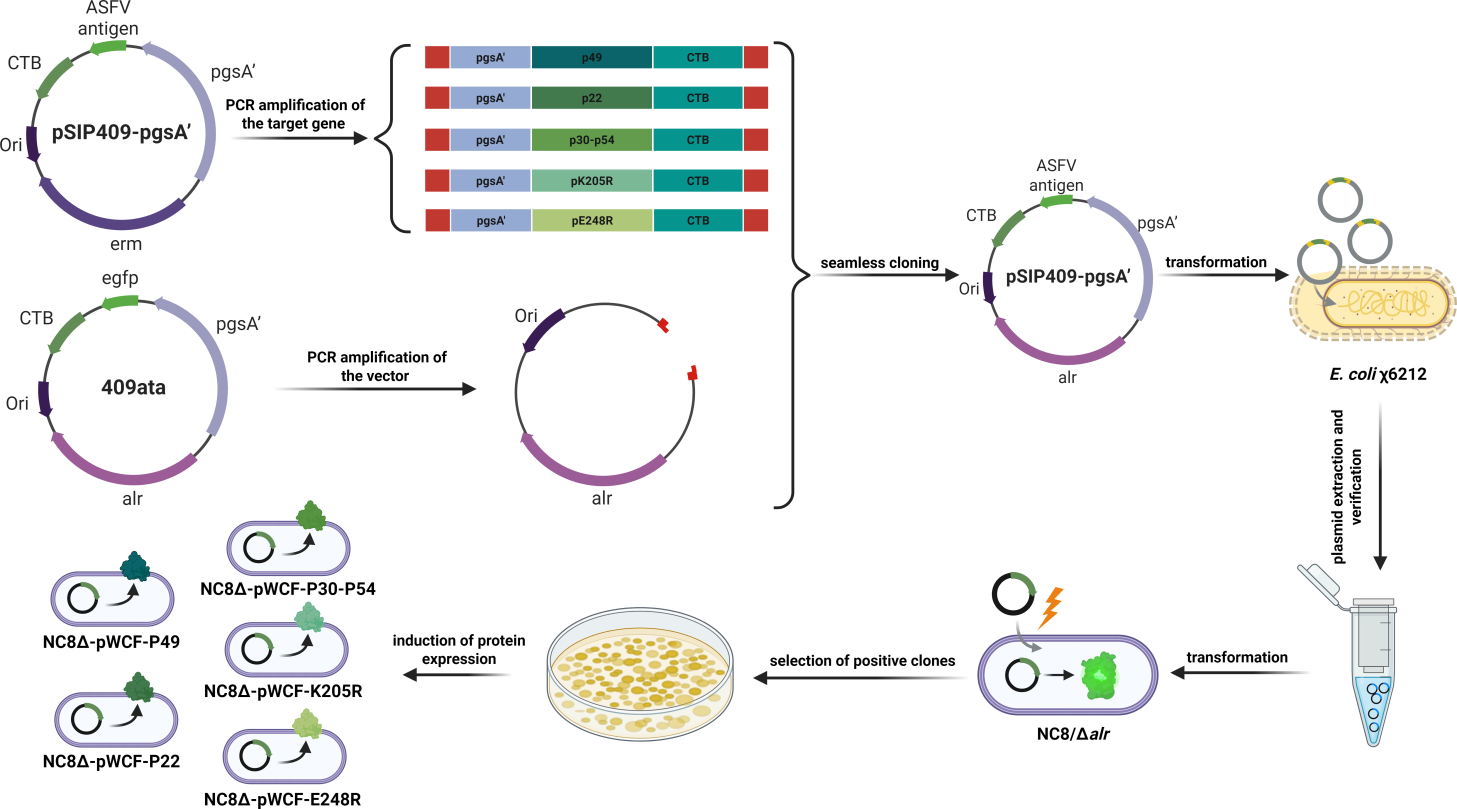
Construction strategy of newly engineered LAB. Seamless cloning primers were used to PCR the fragments of the pSIP409-pgsA′ plasmid containing five ASFV antigen genes and the 409ata plasmid without antibiotic resistance. These fragments were then subjected to seamless cloning and transformed into *E. coli* χ6212. The correctly identified plasmids were subsequently transferred into *L. plantarum* NC8/Δ*alr*, ultimately yielding five newly engineered LAB strains without antibiotic resistance: NC8Δ-pWCF-K205R, NC8Δ-pWCF-E248R, NC8Δ-pWCF-P22, NC8Δ-pWCF-P49, and NC8Δ-pWCF-P30-P54.

**Fig 2:**
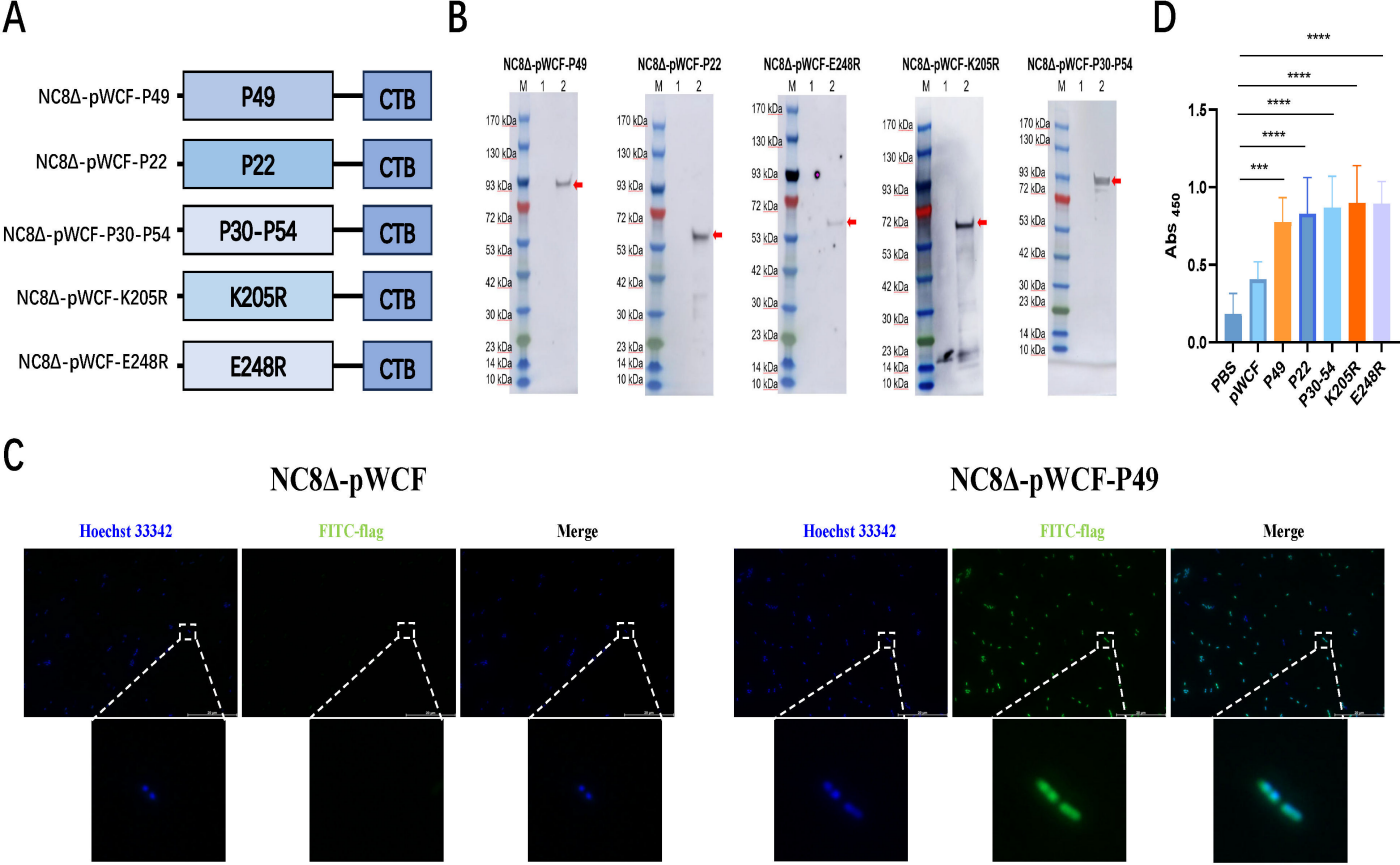
Characterization of ASF vaccine strains. Schematic illustration of the vaccine structure (**A**). Verification of heterologous protein expression. Western blot analysis of total protein extracts from Lactobacillus plantarum expressing ASFV antigenic proteins was performed using mouse anti-FLAG monoclonal antibody. M, protein molecular weight marker; lane 1, NC8Δ-pWCF (empty vector control); lane 2, *L. plantarum* expressing ASFV antigenic proteins. Red arrows indicate the expected molecular sizes of the expressed antigenic proteins. The expected molecular weights for NC8Δ-pWCF-P49, NC8Δ-pWCF-P22, NC8Δ-pWCF-E248R, NC8Δ-pWCF-K205R, and NC8Δ-pWCF-P30-P54 are approximately 90 kDa, 62.1 kDa, 68 kDa, 64.2 kDa, and 88.1 kDa, respectively (**B**). Live bacteria were stained with Hoechst 33342 (blue) and incubated with FITC-conjugated mouse anti-FLAG monoclonal antibody to visualize heterologous protein expression on the bacterial surface (green). Merged images show co-localization. Scale bar, 20 μm (**C**). *In vitro* detection of the ability of fusion antigen protein CTB to target intestinal epithelial cells. The ability of five strains of *L. plantarum* expressing ASFV antigen-CTB fusion proteins to bind to GM1 on intestinal epithelial cells was detected by ELISA. The experiment was independently repeated three times with similar results (**D**). Data were assessed for statistical significance by one-way ANOVA (*n* = 5 mice per group) * P < 0.05 and ** P < 0.01, ** P < 0.01, *** P < 0.001, **** P < 0.0001. NS, not significant.

In this study, the alr gene was employed as an antibiotic-free selection marker, with *L. plantarum* strain NC8Δ serving as the host. Five newly engineered LAB strains were generated, each expressing a distinct ASFV antigen (Fig. 1). To anchor the ASFV antigens on the newly engineered LAB surface, the adjuvant CTB was fused with the ASFV antigen proteins p30 and p54, p49, p22, pK205R, and pE248R, and these fusion constructs were further linked to the truncated anchoring sequence pgsA′ for surface expression. The resulting fusion products were incorporated into the complementary alr plasmid system (409ata) from the laboratory, successfully producing five newly engineered LAB strains: NC8Δ-pWCF-P30-P54, NC8Δ-pWCF-P49, NC8Δ-pWCF-P22, NC8Δ-pWCF-K205R, and NC8Δ-pWCF-E248R (Fig. 2A). Western blot analysis using an anti-Flag tag mouse monoclonal antibody confirmed that NC8Δ-pWCF, NC8Δ-pWCF-K205R (64.2kDa), NC8Δ-pWCF-E248R (68kDa), NC8Δ-pWCF-P22 (62.1kDa), NC8Δ-pWCF-P49 (90kDa), and NC8Δ-pWCF-P30-P54 (88.1kDa) correctly expressed the target proteins (Fig. 2B). These results indicate that the five ASFV plasmids were successfully constructed and expressed in *L. plantarum* strain NC8Δ.

To verify that the ASFV antigen–CTB fusion protein was successfully anchored on the surface of *L. plantarum* strain NC8Δ, the newly engineered LAB strains NC8Δ-pWCF, NC8Δ-pWCF-P30-P54, NC8Δ-pWCF-P49, NC8Δ-pWCF-P22, NC8Δ-pWCF-K205R and NC8Δ-pWCF-E248R were subjected to surface antigen protein visualization using FITC-conjugated mouse anti-FLAG monoclonal antibodies. Fluorescence microscopy revealed bacilli exhibiting green fluorescence across the entire cell surface (Fig. 2C; Fig. S1), indicating that the ASFV antigen–CTB fusion protein was effectively displayed on the bacterial surface.

Furthermore, to assess the targeting capability of the adjuvant–antigen fusion protein, an ELISA was performed. This confirmed that the surface-displayed ASFV–CTB fusion protein could specifically bind to GM1 ganglioside on the surface of mouse intestinal epithelial cells *in vitro* (Fig. 2D). Together, these results demonstrate that the ASFV antigen–CTB fusion protein is not only anchored on *L. plantarum* NC8Δ but also retains its ability to target GM1 ganglioside on intestinal epithelial cells.

### Oral administration of newly engineered LAB stimulates the activation of DCs in mice

**Fig 3:**
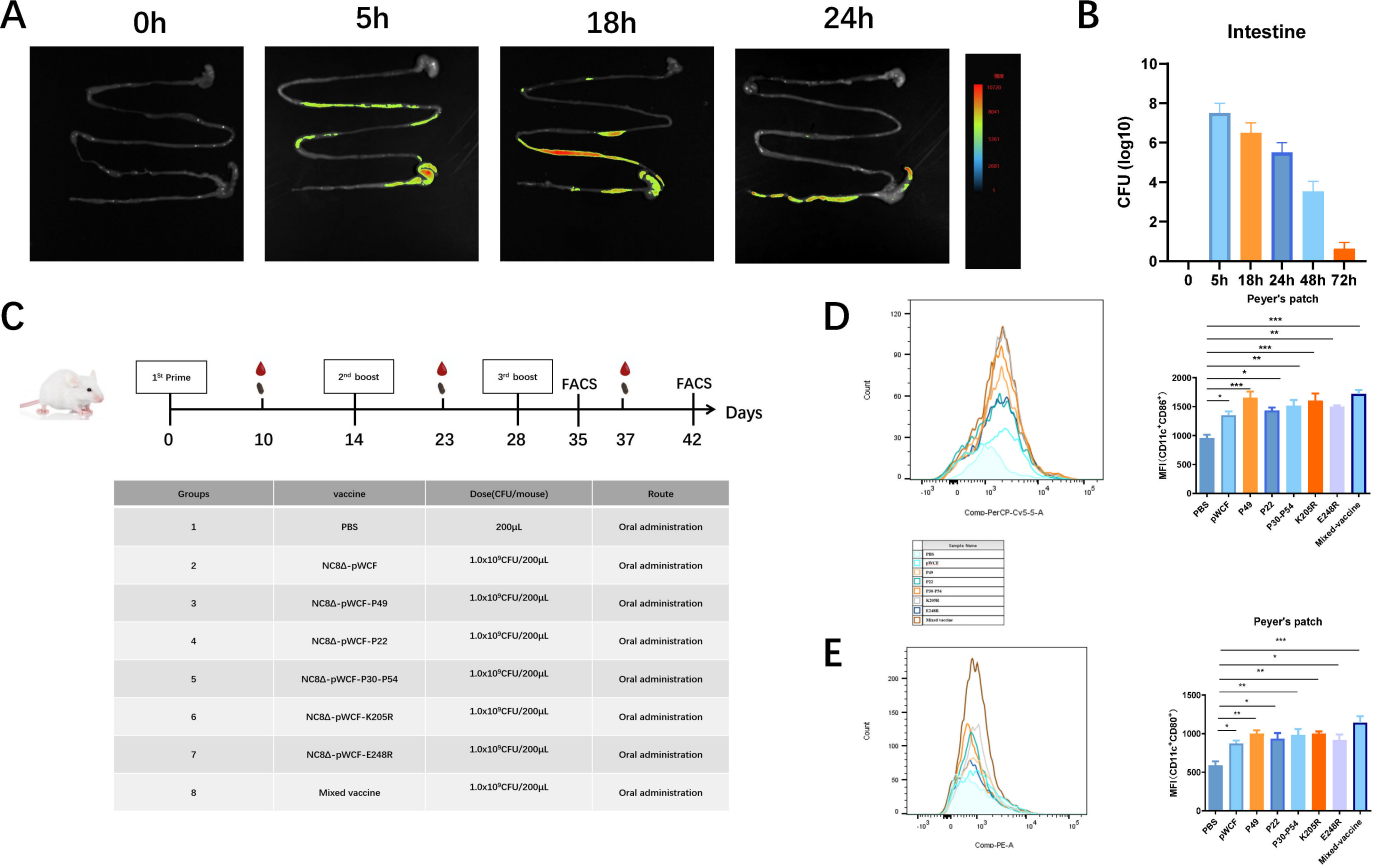
Recombinant lactobacilli targeting intestinal epithelial cells stimulate maturation of DCs. Representative *in vivo* imaging showing the distribution of engineered *L. plantarum* NC8/Δ*alr* expressing EGFP (NC8-EGFP, at a dose of 1 × 10⁹ CFU) in the mouse gastrointestinal tract following oral delivery (**A**). Colonization of engineered *L. plantarum* NC8/Δ*alr* expressing EGFP in the gastrointestinal tract as detected by colony counting (or plate counting) (**B**). Immunization schedule and doses for mice. Six-week-old female mice were immunized three times with 1 × 10⁹ CFU of newly engineered LAB each time, with a 14-day interval between each immunization. Blood and fecal samples were collected 7 days after each immunization. Flow cytometry was performed 5 days and 14 days after the last immunization (**C**). Five days after the third immunization, PPs from mice were collected, and the fluorescence intensity of CD11c^+^CD86^+^ (**D**) and CD11c^+^CD80^+^ (**E**) in the PPs was detected by flow cytometry. Data were assessed for statistical significance by one-way ANOVA (*n* = 5 mice per group) *P < 0.05 and **P < 0.01, **P < 0.01, ***P < 0.001, ****P < 0.0001. NS, not significant.

To evaluate the potential of utilizing *L. plantarum* NC8Δ as a vaccine delivery vehicle, we first investigated the intestinal distribution of NC8 following oral administration in mice. The majority of bacteria were observed to localize within the gastrointestinal (GI) tract, with no detectable presence observed in other tissues (Fig. 3A). Colony counting revealed that *L. plantarum* NC8Δ could persist in the gut for at least 3 days (Fig. 3B). Importantly, bacterial dissemination was strictly limited to the intestinal lumen, with no CFU detected in the heart, liver, spleen, lungs, or kidneys (Fig. S2).

To investigate the immunological effects of the five newly engineered LAB strains, 6-week-old female mice were immunized three times with 1 × 10⁹ CFU of newly engineered LAB per immunization, with a 14-day interval between each dose. Blood and fecal samples were collected seven days following each immunization. Flow cytometry analyses were conducted 5 and 14 days after the final immunization (Fig. 3C). To assess the impact of oral immunization with single-strain and mixed newly engineered LAB on DC activation in mice, DC activation in the Peyer’s Patches (PPs) was evaluated 5 days after the third immunization. Compared with the PBS group, administration of NC8Δ-pWCF-P22 significantly increased the proportion of CD11c^+^CD80^+^ cells (*P* < 0.05). The NC8Δ-pWCF-E248R and NC8Δ-pWCF-P30-54 groups markedly elevated the proportion of CD11c^+^CD80^+^ cells (*P* < 0.01). Notably, the NC8Δ-pWCF-P49, NC8Δ-pWCF-K205R, and mixed newly engineered LAB groups (mixed vaccine group) induced a highly significant increase in the proportion of CD11c^+^CD80^+^ cells in PPs (*P* < 0.001, Fig. 3D). Subsequently, the effects of single-strain and mixed-strain immunization with the newly engineered LAB on the proportion of CD11c^+^CD86^+^ cells were examined. Relative to the PBS group, single-strain immunization significantly increased the proportion of CD11c^+^CD86^+^ cells (*P* < 0.01), whereas the mixed vaccine group produced an extremely significant increase in CD11c^+^CD86^+^ cells (*P* < 0.001, Fig. 3E).

In conclusion, the experimental results demonstrate that both single-strain and mixed-strain immunization with the newly engineered LAB significantly enhance the expression of DC costimulatory molecules CD80 and CD86 in PPs and further indicate that immunization with these LAB strains effectively stimulates DC activation in the PPs of mice.

### Oral immunization with newly engineered LAB elicits effective T-cell responses

**Fig 4:**
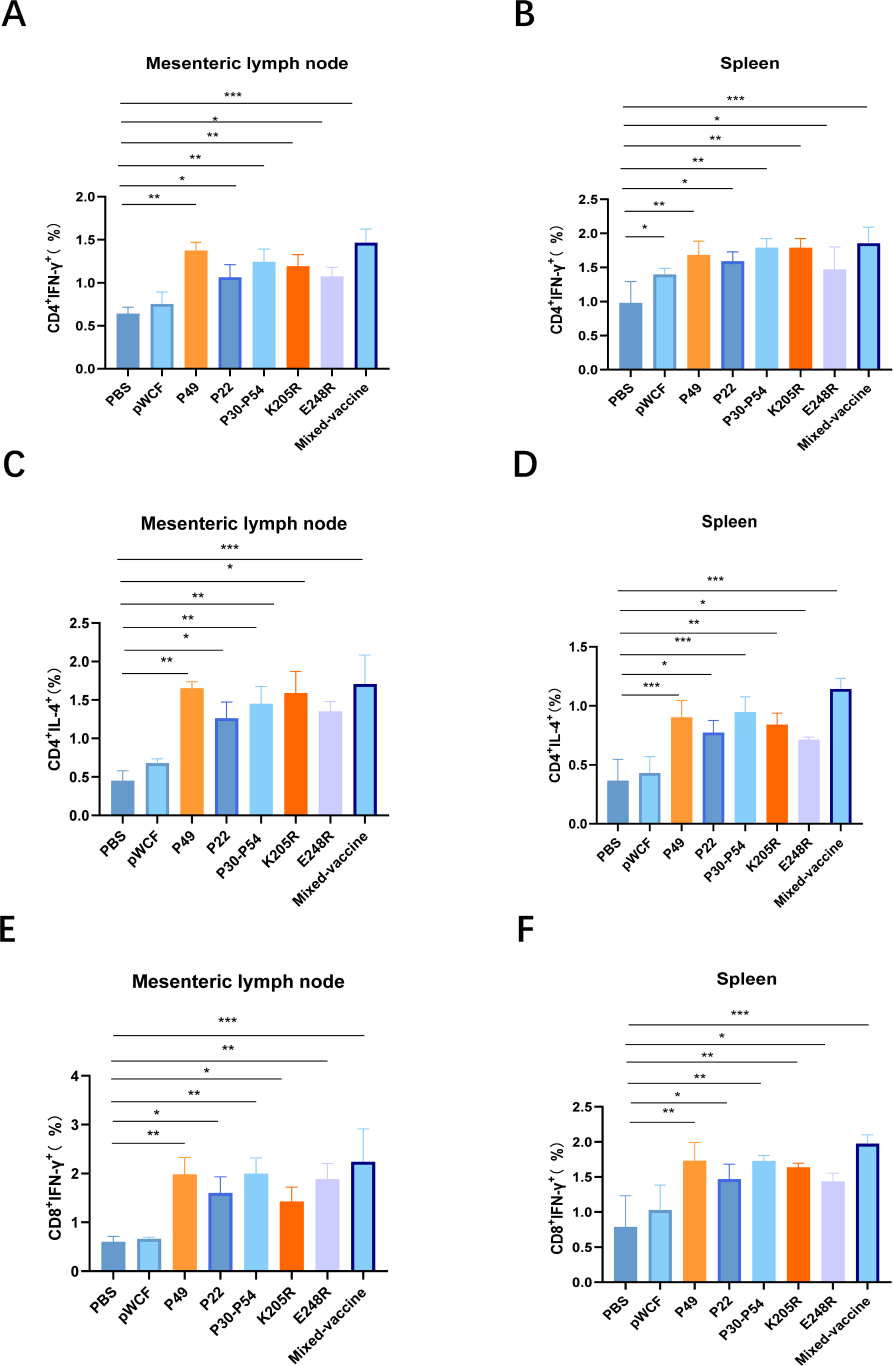
Newly engineered LAB stimulate T-cell activation. Fourteen days after the third immunization, the MLN and spleens of mice were collected, and proportions of CD4^+^IFN-γ^+^ T cells in the MLN (**A**) and spleen (**B**) were detected by flow cytometry. Proportions of CD4^+^IL-4^+^ T cells in MLN (**C**) and spleen (**D**) and CD8^+^IFN-γ^+^ T cells in MLN (**E**) and spleen (**F**) were also detected by flow cytometry. Data were assessed for statistical significance by one-way ANOVA (*n* = 5 mice per group) *P < 0.05 and **P < 0.01, **P < 0.01, ***P < 0.001, ****P < 0.0001. NS, not significant.

To evaluate the effects of oral immunization with single-strain and mixed newly engineered LAB on T-cell activation in mice, intracellular cytokine staining of T cells in the mesenteric lymph nodes (MLN) and spleens was performed 14 days after the third immunization using flow cytometry to assess the cellular immune response. The activation of CD4^+^IFN-γ^+^ T cells, CD8^+^IFN-γ^+^ T cells, and CD4^+^IL-4^+^ T cells in the MLN and spleens of mice immunized with the newly engineered LAB was analyzed. Specifically, the activation of CD4^+^IL-4^+^ T cells in the MLN and spleens was first assessed by flow cytometry. In the MLN, compared with the PBS group, the proportions of CD4^+^IFN-γ^+^ T cells were increased in the NC8Δ-pWCF-P22 and NC8Δ-pWCF-E248R groups (*P* < 0.05). The NC8Δ-pWCF-P49, NC8Δ-pWCF-P30-P54, and NC8Δ-pWCF-K205R groups showed a more pronounced increase in CD4^+^IFN-γ^+^ T-cell proportions (*P* < 0.01). Notably, the mixed vaccine group induced a highly significant increase in the proportion of CD4^+^IFN-γ^+^ T cells (*P* < 0.001, Fig. 4A). Consistent results were observed in the spleen (Fig. 4B; Fig. S3).

Subsequently, the activation of CD4^+^IL-4^+^ T cells in the MLN and spleens of mice immunized with the newly engineered LAB was assessed by flow cytometry. In the MLN, compared with the PBS group, the proportions of CD4^+^IL-4^+^ T cells were increased in the NC8Δ-pWCF-P22 and NC8Δ-pWCF-E248R groups (*P* < 0.05). More pronounced increases were observed in the NC8Δ-pWCF-P49, NC8Δ-pWCF-P30-P54, and NC8Δ-pWCF-K205R groups (*P* < 0.01). Notably, the mixed vaccine group induced a highly significant elevation in the proportion of CD4^+^IL-4^+^ T cells (*P* < 0.001, Fig. 4C). Similar trends were observed in the spleen, where the NC8Δ-pWCF-P49, NC8Δ-pWCF-P30-P54, and mixed vaccine groups exhibited extremely significant increases in CD4^+^IL-4^+^ T-cell proportions (*P* < 0.001, Fig. 4D; Fig. S4).

Finally, the activation of CD8^+^IFN-γ^+^ T cells in the MLN and spleens of mice immunized with the newly engineered LAB was assessed using flow cytometry. In the MLN, the results showed that, compared with the PBS group, the proportions of CD8^+^IFN-γ^+^ T cells increased in the NC8Δ-pWCF-P22 and NC8Δ-pWCF-K205R groups (*P* < 0.05). Moreover, the NC8Δ-pWCF-P49, NC8Δ-pWCF-P30-P54, and NC8Δ-pWCF-E248R groups exhibited a significant increase in CD8^+^IFN-γ^+^ T-cell proportions compared with the PBS group (*P* < 0.01). Notably, the mixed vaccine group produced an extremely significant increase in CD8^+^IFN-γ^+^ T-cell proportions (*P* < 0.001, Fig. 4E). Similar patterns were observed in the spleen (Fig. 4F; Fig. S5).

These findings indicate that immunization with both single-strain and mixed Lactobacillus strains markedly enhances the capacity of T cells in the MLN and spleen to secrete cytokines, with the mixed-strain newly engineered LAB showing the most pronounced effect. Overall, oral immunization with a mixed-strain, newly engineered LAB vaccine effectively modulates the host immune response, promoting viral clearance.

### Oral immunization with newly engineered LAB promotes the transcription and expression of related cytokines

**Fig 5:**
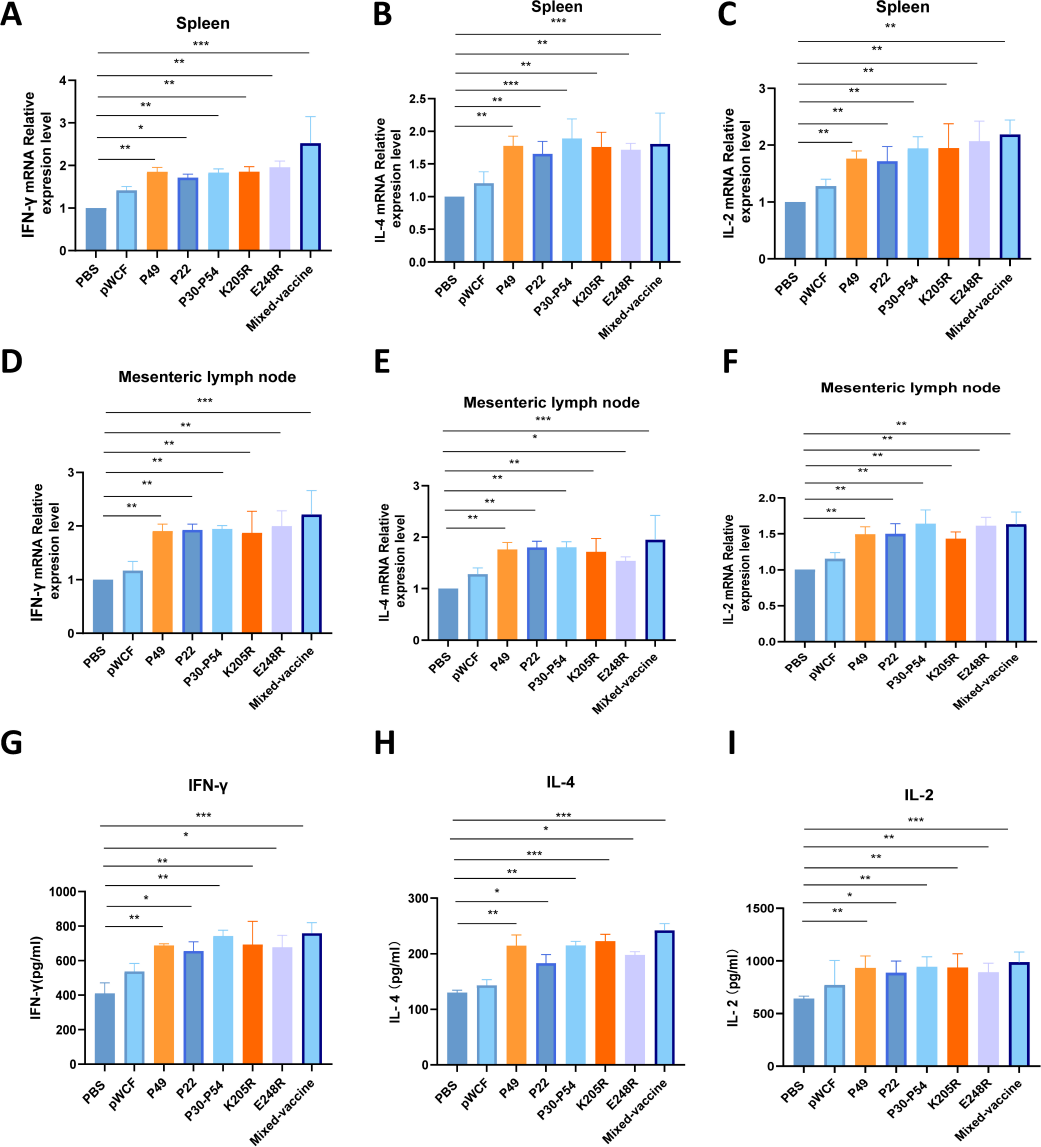
Effects of newly engineered LAB on transcription levels of antiviral cytokines in spleen and MLN, and the levels of cytokines in the serum of mice were investigated. RNA was extracted from the spleen and MLN of mice immunized with the newly engineered LAB. The transcription levels of IFN-γ (**A**), IL-4 (**B**), and IL-2 (**C**) in the spleen, and IFN-γ (**D**), IL-4 (**E**), and IL-2 (**F**) in the MLN were observed. The effects of newly engineered LAB on the expression levels of related cytokines in the serum of mice were also examined. Expression levels of IFN-γ (**G**), IL-4 (**H**), and IL-2 (**I**) in the serum of mice immunized with newly engineered LAB were detected by ELISA. Data were assessed for statistical significance by one-way ANOVA (*n* = 5 mice per group) *P < 0.05 and **P < 0.01, **P < 0.01, ***P < 0.001, ****P < 0.0001. NS, not significant.

To evaluate the effects of oral immunization with single-strain and mixed newly engineered LAB on antiviral cytokines in the spleen, MLN, and serum of mice, quantitative fluorescence was used to measure transcription levels of interleukin 4 (IL-4), IL-2, and gamma interferon (IFN-γ) in the spleen and MLN. The results demonstrated that single-strain newly engineered LAB significantly elevated the transcription levels of IL-4, IL-2, and IFN-γ in both tissues. Notably, mice immunized with mixed Lactobacillus strains exhibited a markedly higher increase in the transcription levels of these three key cytokines in the MLN and spleen (*P* < 0.001, Fig. 5A, B, D and E). These findings suggest that oral administration of mixed newly engineered LAB enhances the activation of immune cells in the MLN and spleen, further corroborating the flow cytometry results.

The levels of IFN-γ, IL-4, and IL-2 in the mouse circulatory system were measured using ELISA. The results indicated that immunization with newly engineered LAB enhanced the expression of all three cytokines in mouse serum. Notably, compared with the PBS group, oral administration of mixed newly engineered LAB produced an extremely significant increase in IFN-γ, IL-4, and IL-2 levels (*P* < 0.001, Fig. 5G through I). These findings suggest that both single-strain and mixed newly engineered LAB enhance the capacity of immune cells to secrete cytokines.

### Oral administration of newly engineered LAB induces B-cell activation in mice

**Fig 6:**
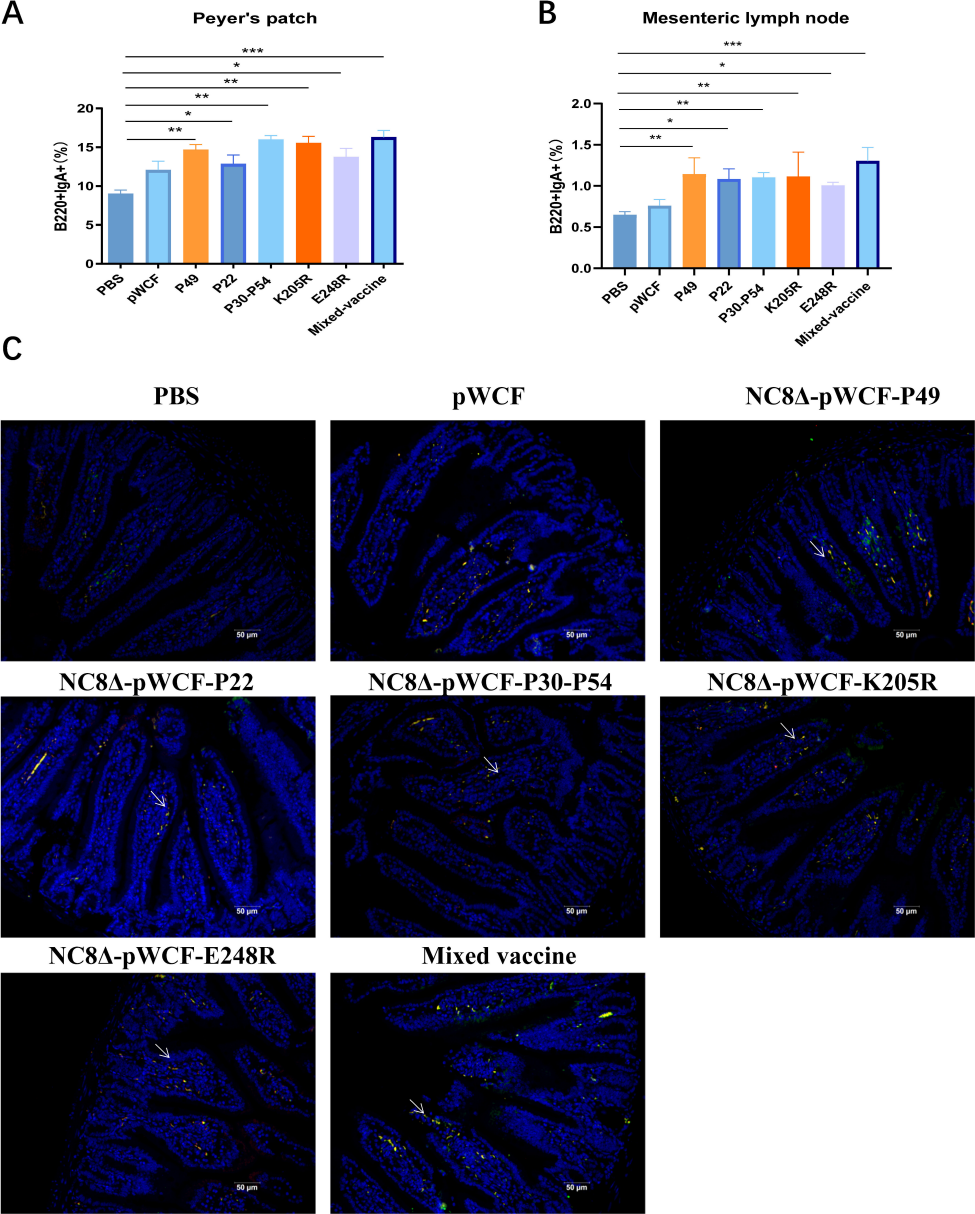
Newly engineered LAB stimulate B-cell activation. Five days after the third immunization, PPs and MLN were collected from mice, and proportions of B220^+^IgA^+^ cells in the PP (**A**) and MLN (**B**) were detected by flow cytometry. The activation level of B cells in the mouse duodenum was detected by immunofluorescence. Cells were labeled with anti-mouse B220-PE mAb and anti-mouse IgA-FITC mAb, and B220^+^IgA^+^ cells appeared yellow under the fluorescence microscope (**C**). The scale bar in the duodenum represents 50 μm. Data were assessed for statistical significance by one-way ANOVA (*n* = 5 mice per group) *P < 0.05 and **P < 0.01, **P < 0.01, ***P < 0.001, ****P < 0.0001. NS, not significant.

To evaluate the effects of oral immunization with single-strain and mixed newly engineered LAB on B-cell activation in mice, the activation status of B cells in PPs was assessed using flow cytometry. The results showed that, compared with the PBS group, the proportion of B220^+^IgA^+^ B cells increased in the NC8Δ-pWCF-P22 and NC8Δ-pWCF-E248R groups (*P* < 0.05), while the NC8Δ-pWCF-P49, NC8Δ-pWCF-P30-P54, and NC8Δ-pWCF-K205R groups exhibited a significant increase in B220^+^IgA^+^ B cells (*P* < 0.01). Notably, oral administration of mixed Lactobacillus strains led to an extremely significant increase in the proportion of B220^+^IgA^+^ B cells in the intestinal mucosal effector sites (*P* < 0.001, Fig. 6A). Similar results were observed in the MLN (Fig. 6B; Fig. S6). These findings indicate that oral immunization with both single-strain and mixed Lactobacillus strains enhances the proliferation and activation of B cells in intestinal mucosal effector sites.

The expression of B cells in the lamina propria of the duodenum, a key mucosal immune effector site, was assessed using immunofluorescence. Following immunization with newly engineered LAB, both single-strain and mixed newly engineered LAB increased the number of B220^+^IgA^+^ B cells in the duodenum compared with the PBS group (Fig. 6C).

### Oral administration of newly engineered LAB increases the levels of specific antibodies

**Fig 7:**
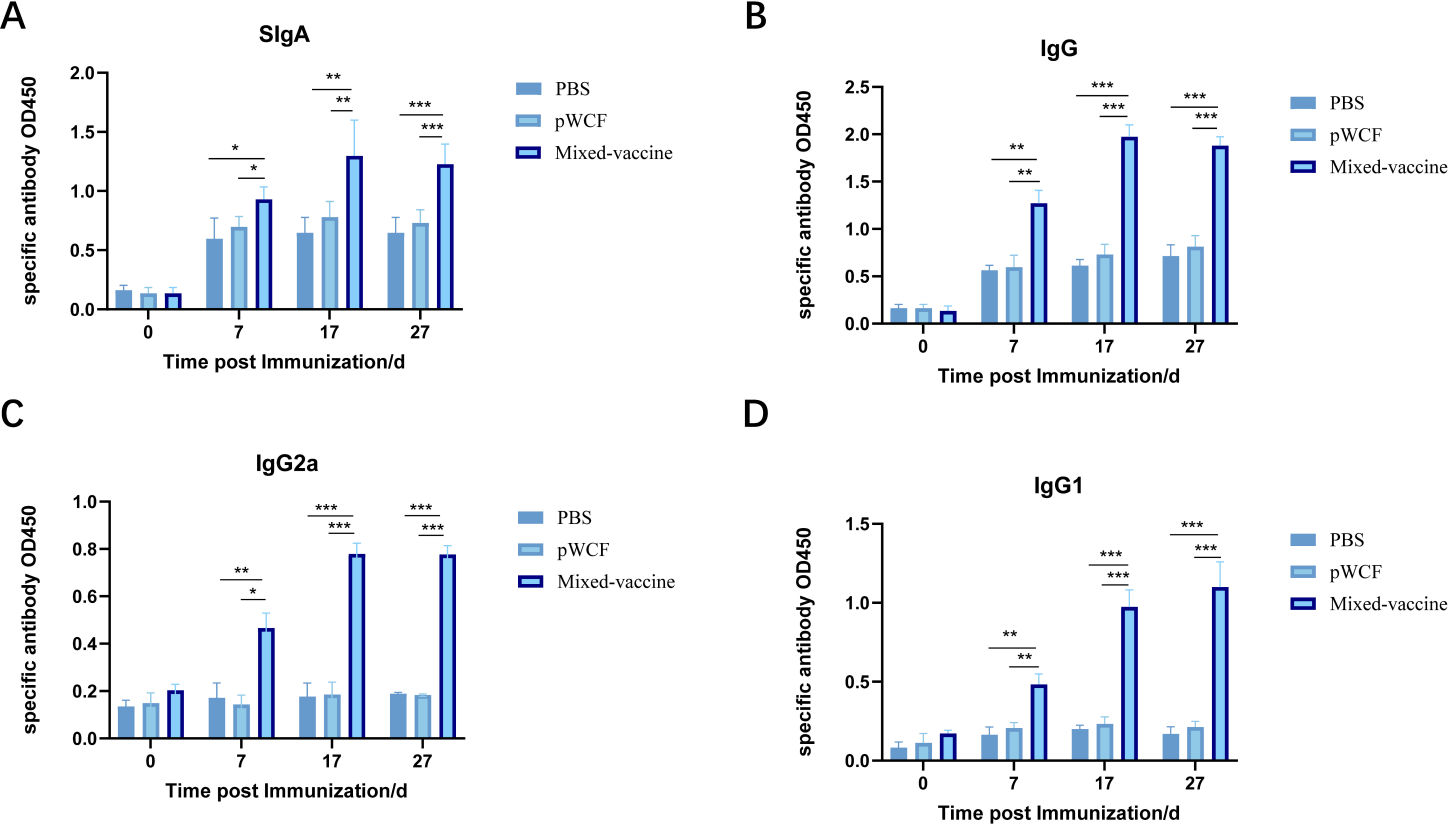
Effect of newly engineered LAB on expression levels of antigen-specific antibodies in mouse serum and feces. Expression levels of antigen-specific SIgA in feces (**A**) and IgG (**B**), IgG2a (**C**), and IgG1 (**D**) in serum of mice immunized with the newly engineered LAB were detected by ELISA. Data were assessed for statistical significance by one-way ANOVA (*n* = 5 mice per group) *P < 0.05 and **P < 0.01, ***P < 0.001, ****P < 0.0001. NS, not significant.

To assess the capacity of mice to generate ASF antigen-specific humoral and mucosal immune responses following immunization, the expression levels of antigen-specific IgA and IgG in serum and feces were measured 14 days after the third immunization. To evaluate the mucosal immune response, the secretion levels of antigen-specific IgA in feces were first analyzed. Compared with the PBS group, oral administration of mixed Lactobacillus strains significantly elevated the levels of specific SIgA in the fecal supernatant (*P* < 0.001, Fig. 7A). These results indicate that immunization with newly engineered LAB enhances humoral immunity at mucosal sites.

The effects of oral administration of mixed *L. plantarum* strains on the levels of antigen-specific IgG and its subtypes in mouse serum were further evaluated by ELISA. Compared with the PBS group, oral administration of mixed Lactobacillus strains significantly increased the levels of antigen-specific IgG, as well as its subtypes IgG1 and IgG2a, in mouse serum (*P* < 0.001, Fig. 7B through D). Collectively, these results indicate that oral administration of mixed Lactobacillus strains effectively stimulates the host immune response, leading to the production of specific antibodies.

## DISCUSSION

To date, ASF remains a significant threat to the global swine industry, and vaccination is still considered the most effective strategy for controlling ASF outbreaks (19). Previous studies have indicated that vaccination can provide a degree of protection against ASFV. In this study, we demonstrated that oral administration of a cocktail vaccine, composed of *L. plantarum* NC8 strains expressing p30, p54, p49, p22, pK205R, and pE248R, effectively elicited both systemic and mucosal immune responses specific to the selected antigens.

The mucosa serves as the primary route of entry for ASFV into the host organism (20, 21). Research has demonstrated that IN immunization with a replication-incompetent type-2 adenovirus vector vaccine expressing ASFV antigens (p30, p54, CD2v, p72, and the p72 chaperone) effectively induces both systemic and mucosal immune responses in mice and pigs, providing robust protection against circulating ASFV strains in farmed pigs (11). This underscores the critical role of the mucosa in ASFV invasion and highlights the importance of establishing a strong immune barrier at the mucosal level to prevent infection. However, a significant challenge remains: although ASFV antigen proteins exhibit considerable antigenicity and immunogenicity, their protective efficacy alone is insufficient to confer adequate immune protection when employed as vaccine components. To enhance vaccine efficacy, we explored the strategy of fusing adjuvants to these antigen proteins, aiming to boost their immunogenicity and develop a more effective ASFV vaccine. Supporting this approach, prior studies have shown that fusion expression of antigen proteins with adjuvants can markedly enhance vaccine-induced protection (22). Among various potential adjuvants, CTB has garnered particular attention due to its unique immunomodulatory properties (23). CTB can activate DCs, thereby inducing long-lasting protective immunity (24, 25). Nevertheless, its application in ASF vaccines has not yet been reported. In this study, we fused CTB with multiple ASFV antigen proteins to enhance their immunogenicity (Fig. 2A). The CTB–ASFV antigen fusion constructs were successfully generated and expressed using the complementary alr plasmid system (409ata), which does not require antibiotic resistance markers, minimizing potential environmental impact and demonstrating high environmental safety (Fig. 2B). It is worth mentioning that the use of live recombinant bacteria as veterinary vaccines faces stringent regulatory restrictions in many jurisdictions, including the European Union, the United States, and China. These regulations primarily address concerns regarding horizontal gene transfer (HGT), environmental persistence, and the potential dissemination of antibiotic resistance markers (26). To address these biosafety challenges, the present study employed an auxotrophic *Lactobacillus* chassis (Δ*alr*) that requires exogenous alanine for survival (27). This genetic containment strategy ensures that the recombinant strains cannot replicate or persist in the natural environment or animal gastrointestinal tract without supplementation, effectively eliminating the risk of environmental persistence. Furthermore, the use of an antibiotic resistance-free *alr* complementation selection system (replacing conventional antibiotic markers) further reduces the potential risk of horizontal gene transfer. While the current study establishes proof-of-concept, the strategy of employing Generally Recognized As Safe (GRAS) organisms (*Lactobacillus* spp.) combined with irreversible auxotrophic containment is one of the measures for meeting future regulatory requirements. The ASFV antigen–CTB fusion protein displayed on the surface of *L. plantarum* NC8 exhibited strong binding affinity for GM1 (Fig. 2D). Given CTB’s high affinity for GM1, this fusion protein can efficiently target GM1 on the intestinal mucosal surface, prolonging the vaccine’s residence time in the gut. This extended retention not only enhances interactions with the gut mucosal immune system but also promotes a more robust mucosal immune response. By strengthening mucosal immunity, the vaccine can more effectively establish an immune barrier on the mucosal surface, resisting ASFV invasion and demonstrating significant antiviral effects. Furthermore, the ASFV antigen–CTB fusion protein displayed on *L. plantarum* NC8 markedly stimulated DC activation in PPs (Fig. 3D and E). Collectively, these findings indicate that fusing ASFV antigen proteins with CTB can elicit a stronger immune response against ASFV antigens.

In ASFV vaccine research, IgA, an antibody generated by mucosal immune responses, plays a crucial role in preventing pathogen infections in mucosal tissues (28). However, compared with oral vaccines, injectable vaccines often have limited capacity to induce mucosal immunity and confer protection against mucosal infections (29). In contrast, oral delivery of antigens using *L. plantarum* as a carrier provides a novel and safe vaccine platform. Specifically, *L. plantarum* displaying surface-anchored CTB–ASFV antigen proteins can interact with mucosal epithelial cells, and this adhesion promotes the induction of adaptive immune responses (30). This effect is evident in the oral administration of a mixture of newly engineered LAB, which significantly enhances the expression of antigen-specific IgA in the gut and increases B-cell activation in PPs and MLNs (Fig. 6A and B). Interestingly, compared to the PBS group, the empty vector strain also significantly increased the activation of dendritic cells and B cells in the PPs (Fig. 3D, E and 6A). This phenomenon aligns with the inherent immunostimulatory properties of LAB. Extensive literature indicates that even LAB without heterologous antigens (or those transformed with empty vectors) contain cell wall components (such as peptidoglycan and lipoteichoic acid) that can act as pathogen-associated molecular patterns (31). These components non-specifically activate the innate immune system through pathways like Toll-like receptors, leading to mild, broad-spectrum inflammatory cytokine production and immune cell recruitment (32). This stands in stark contrast to the completely inert nature of PBS and explains why the empty vector group induced baseline-level immune activation. Building upon this inherent immune activation, oral administration of the newly engineered lactic acid bacteria mixture further stimulates. Following oral administration, the newly engineered LAB mixture stimulates the gut mucosa to produce higher levels of antigen-specific IgA, which binds to ASFV antigens and blocks viral invasion. Simultaneously, the LAB mixture activates B cells in PPs, promoting their differentiation into plasma cells and further augmenting IgA production, thereby reinforcing mucosal immune protection (Fig. 7A).

In the immune response to ASFV infection, mucosal immunity plays an important role; however, CD4^+^ and CD8^+^ T cells also exert critical functions (33). Research has demonstrated that ASFV-specific antibodies alone are insufficient to prevent ASFV infection (34). Instead, CD4^+^ and CD8^+^ T lymphocyte subsets are essential for protective immunity against ASFV. CD4^+^ T cells primarily regulate immune responses by secreting cytokines that activate other immune cells, including macrophages and B cells, thereby enhancing overall immune defense. Notably, IFN-γ promotes antigen presentation and subsequently activates CD8^+^ T cells. Furthermore, CD4^+^ T cells facilitate B-cell proliferation and antibody production through cytokines such as IL-4, which can neutralize ASFV and prevent further host cell infection. Our results show that oral immunization with a newly engineered LAB mixture significantly increases IL-4 secretion by CD4^+^ T cells in the MLN and spleen of mice while also enhancing IFN-γ production by both CD4^+^ and CD8^+^ T cells (Fig. 4A, B, E and F). This multifaceted immune activation contributes to a robust protective response in immunized mice. Specifically, IFN-γ, as a bioactive molecule, can inhibit viral replication, whereas IL-4, produced by Th2 cells, not only activates cytotoxic T cells but also plays a pivotal role in humoral immune responses and antibody production (35). Additionally, IL-2, primarily secreted by activated T cells, significantly stimulates lymphocyte proliferation and differentiation, thereby amplifying T-cell activation and cytokine secretion (36). The synergistic effects of these cytokines enhance cellular immunity while promoting comprehensive humoral responses. Given their critical roles, IFN-γ and IL-4 levels are commonly used as key indicators to evaluate vaccine immunogenicity (37). In our study, cytokine analysis in mouse serum revealed that oral administration of the LAB mixture markedly increased IFN-γ, IL-4, and IL-2 expression, and transcriptional analysis confirmed significant upregulation of these cytokines in the spleen and MLN (Fig. 5). Collectively, these findings indicate that the newly engineered LAB mixture effectively induces both cellular and humoral immune responses, providing comprehensive protection. In summary, CD4^+^ and CD8^+^ T cells work synergistically during the immune response to ASFV infection. CD4^+^ T cells orchestrate immune regulation through cytokine secretion and activation of other immune cells, while CD8^+^ T cells directly eliminate infected cells, together conferring robust protection against ASFV invasion.

It is well established that IgG antibodies play a critical role in enhancing the body’s antiviral defenses. Th1-type immune responses are characterized by the production of IgG2a antibodies and the recruitment of cytotoxic T lymphocytes (CTLs), macrophages, and natural killer (NK) cells, thereby strengthening cellular immunity. In contrast, Th2-type responses are marked by the production of IgG1 antibodies and primarily promote humoral immunity. Both response types are essential for the effective clearance of viral infections (38). Our findings further support this, showing that oral administration of the newly engineered LAB mixture significantly increases the levels of total IgG as well as its subtypes, IgG1 and IgG2a, in mouse serum (Fig. 7B through D). Notably, this mixture markedly elevates IgG2a levels, indicative of Th1-type responses, while also significantly boosting IgG1 levels, reflective of Th2-type responses. This dual activation suggests that the newly engineered LAB mixture can simultaneously stimulate both cellular and humoral immune responses. Consequently, the oral vaccination strategy using this newly engineered LAB mixture shows considerable potential for broad protection against ASFV, enhancing antiviral capacity and providing comprehensive immune defense through multiple mechanisms to effectively prevent infection.

### Conclusion

**Fig 8:**
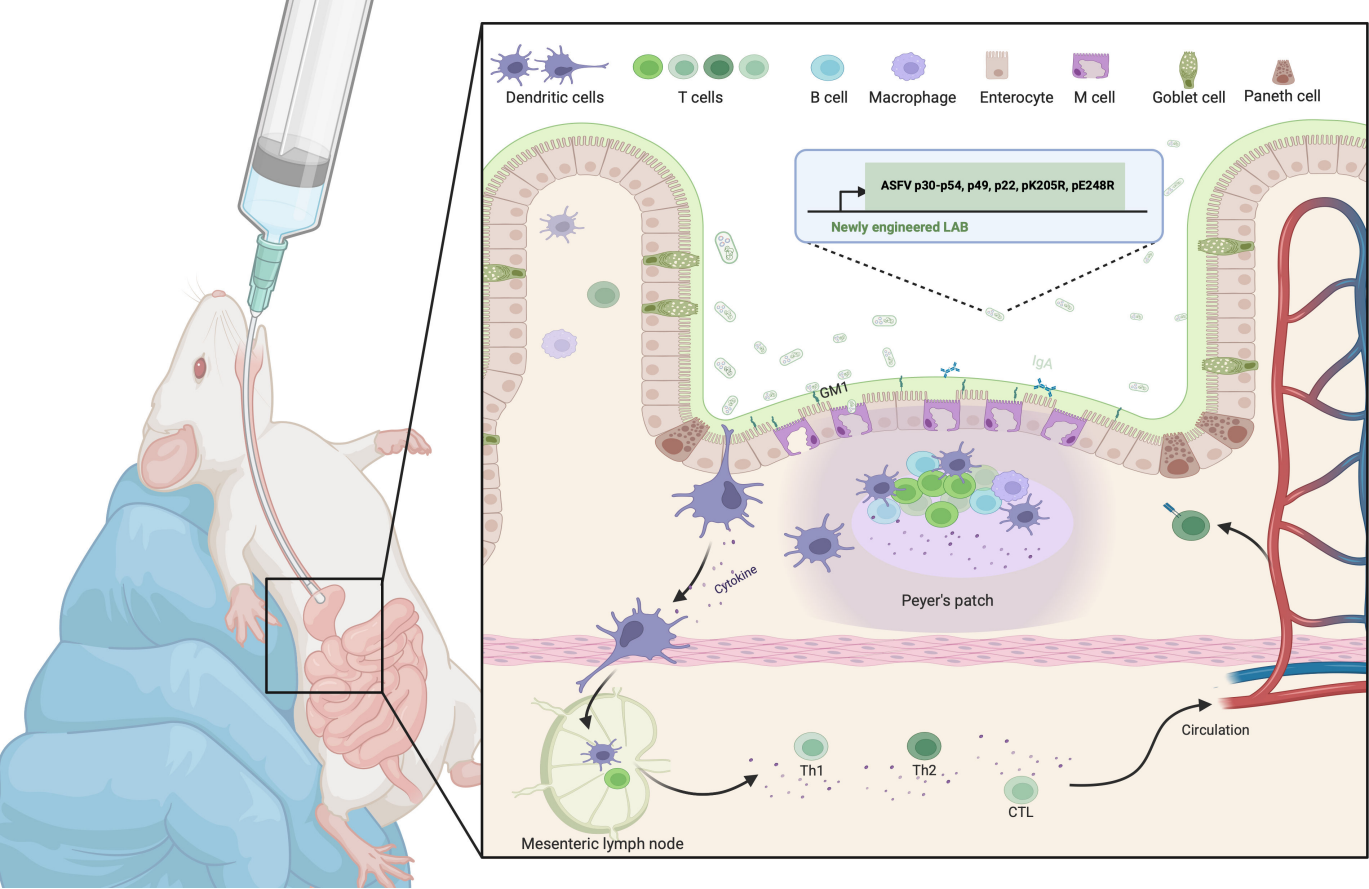
Oral ASFV vaccine based on newly engineered LAB. The CTB was genetically fused with ASFV antigen proteins (p30, p54, p49, p22, pK205R, and pE248R) and successfully anchored on the surface of the antibiotic-free selection system of *L. plantarum* NC8, resulting in the construction of five newly engineered LAB strains. After oral administration of these newly engineered LAB strains to mice in a mixed formulation, the CTB adjuvant anchored on the surface of the engineered LAB interacted with ganglioside GM1 on the surface of intestinal epithelial cells and was recognized and taken up by DCs in the lamina propria. The newly engineered LAB strains, rich in pathogen-associated molecular patterns (PAMPs), rapidly matured the DCs, which then migrated to the MLN to complete the presentation of ASFV antigens. This process effectively activated antigen-specific CTLs, Th cells, and B cells, thereby inducing a specific immune response against ASFV. Graphical illustrations were made with Biorender.com.

In conclusion, our study provides strong evidence supporting the potential of newly engineered LAB as a preventive strategy against ASFV infection. Our results demonstrate that oral administration of the newly engineered LAB mixture effectively stimulates mucosal, humoral, and cellular immune responses in mice. These findings highlight the promise of newly engineered LAB as a versatile platform for antiviral vaccine development (Fig. 8).

## MATERIALS AND METHODS

### Experimental animals

Five-week-old female BALB/c mice were obtained from Huafu Kang Biotechnology Company (Beijing, China) and quarantined for a 7-day observation period prior to experimentation. Following acclimatization, the mice were randomly divided into groups and maintained under specific pathogen-free (SPF) conditions with unrestricted access to food and water. All experimental procedures were performed in strict accordance with national guidelines for the care and use of laboratory animals. Ethical approval for the study was granted by the Animal Ethics Committee of Jilin Agricultural University and the Institutional Life Sciences Ethics Committee.

### Construction of recombinant lactobacillus

The ASFV genes p30, p54, p49, p22, pK205R, and pE248R, derived from the ASFV-SY18 strain (GenBank accession number: MH766894.1), were each fused with the CTB gene (GenBank accession number: AAD51360.1), and a Flag-tag (amino acid sequence: DYKDDDDK) was subsequently introduced at the N-terminus of the resulting fusion protein. Codon optimization for *L. plantarum* was performed by Sangon Biotech (China), and the optimized sequences were subsequently cloned into the pSIP409-pgsA’ vector, generating five recombinant plasmids, each designed to express a specific ASFV antigen. Using specific primers and IN-Fusion cloning technology, the ErmL erythromycin resistance gene in these plasmids was replaced by asd-alr, a fusion gene encoding aspartate β-semialdehyde dehydrogenase and alanine racemase (alr). The resulting plasmids were first introduced into the asd-deficient *Escherichia coli* strain χ6212 for amplification and then transformed into the alr-deficient *L. plantarum* NC8 strain. This process yielded five recombinant *L. plantarum* strains, each expressing a distinct ASFV antigen, designated as NC8Δ-pWCF-P49, NC8Δ-pWCF-P22, NC8Δ-pWCF-K205R, NC8Δ-pWCF-P30-P54, and NC8Δ-pWCF-E248R, respectively.

All strains and plasmids used in this study are summarized in Table S1. Oligos are listed in Table S2. The amino acid sequences of proteins expressed by the constructed plasmids are listed in Table S3.

### Western blot analysis

Recombinant Lactobacillus strains were cultured overnight at 30°C in MRS broth. Following incubation, the cultures were centrifuged at 12,000 × *g* for 2 minutes at 4°C to collect the bacterial cells. The supernatant proteins were then transferred onto a nitrocellulose (NC) membrane, which was subsequently blocked with 5% skim milk for 2 hours to prevent nonspecific binding. The membrane was incubated overnight at 4°C with an anti-Flag tag mouse monoclonal antibody (Beyotime, 1:5,000 dilution). After washing, it was further incubated for 1 hour at room temperature with an HRP-conjugated goat anti-mouse IgG secondary antibody (Beyotime, 1:10,000 dilution). Finally, the membrane was washed five times with TBST and visualized using the Bio-Rad ChemiDoc MP chemiluminescent imaging system (USA).

### Indirect immunofluorescence assay

The cultured Lactobacillus cells were induced overnight using a final concentration of 50 ng/mL SppIP inducer peptide, synthesized by Sangon Biotech (China). After induction, the cells were washed three times with PBS, and the resulting pellet was collected. The pellet was then blocked with 1% BSA on a shaker at 4°C for 1 hour. Subsequently, the *L. plantarum* cells expressing Flag-tagged antigen proteins on their surface were incubated overnight at 4°C with an anti-Flag tag mouse monoclonal antibody (Beyotime). After incubation, the cells were washed twice with PBS, resuspended in FITC-labeled goat anti-mouse IgG (Beyotime), and incubated on a shaker at 4°C for 2 hours. Following another wash step, the samples were examined and photographed using a Leica upright fluorescence microscope (Germany).

### GM1 ganglioside-binding assay by ELISA

An ELISA plate was coated overnight with 1 μg/mL GM1 ganglioside (Sigma). The plate was then blocked with 2% BSA at 37°C for 1 hour. Similarly, Lactobacillus cells expressing CTB–ASFV antigen fusion proteins were blocked with 2% BSA at 37°C for 1 hour. The bacteria were subsequently added to the wells and incubated. Each well was then treated with an anti-Flag tag mouse monoclonal antibody (Beyotime) for 1 hour, followed by the addition of 100 μL HRP-conjugated goat anti-mouse IgG (Beyotime) and further incubation at 37°C for 1 hour. After thorough washing three times with PBST, TMB substrate was added, and the optical density at 450 nm (OD_450_) was measured using a BioTek 800 TS Absorbance Reader equipped with Gen5 software (USA).

### In *vivo* fluorescence imaging and CFU analysis

Mice were orally gavaged with recombinant lactobacilli harboring the pSIP409-egfp plasmid (containing the erythromycin resistance gene) at a dose of 10^9^ CFU per 100 μL. At specific time points, *in vivo* fluorescence imaging of the gastrointestinal tract, heart, liver, spleen, lungs, and kidneys was performed using an *in vivo* imaging system (Germany). Intestinal contents (feces) were collected, and tissues were mechanically homogenized, resuspended in PBS, serially diluted, and plated onto MRS agar supplemented with erythromycin and 2% alanine. After overnight incubation at 37°C, the number of viable bacteria in the intestine was determined by CFU enumeration.

### Mouse immunization

Six-week-old female BALB/c mice were assigned to eight groups: the PBS group, the empty vector NC8Δ-pWCF group, the NC8Δ-pWCF-P30-P54 group, the NC8Δ-pWCF-P49 group, the NC8Δ-pWCF-P22 group, the NC8Δ-pWCF-K205R group, the NC8Δ-pWCF-E248R group, and a mixed-vaccine group containing a combination of the five newly engineered LAB strains. The immunization regimen consisted of three immunizations, each administered over three consecutive days, with 1 × 10⁹ CFU of the respective newly engineered LAB strain per dose. A 14-day interval was maintained between immunizations. Serum and fecal samples were collected 1 week after each immunization to assess antigen-specific antibody and cytokine levels, and 2 weeks after the final immunization, the activation levels of immune cells were evaluated.

### Flow cytometry

PPs were collected from mice 5 days after the final immunization, while MLN and spleens were harvested 14 days post-final immunization. Single-cell suspensions were prepared from these tissues, and subsequent procedures were carried out following the established laboratory protocol (39). The activation levels of dendritic cells (DCs) in PPs, B cells in PPs and MLN, and the secretion levels of IFN-γ and IL-4 by CD4^+^ and CD8^+^ T cells in the spleen and MLN were analyzed using the BD LSRFortessa multicolor flow cytometry system (USA). The antibodies employed included the following: anti-mouse CD80-PerCP-Cy5.5 mAb, anti-mouse CD86-PE mAb, anti-mouse CD11c-APC mAb, anti-mouse B220-PE mAb, anti-mouse IgA-FITC mAb, anti-mouse CD3-AF700 mAb, anti-mouse CD4-PerCP-Cy5.5 mAb, anti-mouse CD8-FITC mAb, anti-mouse IFN-γ-PE mAb, and anti-mouse IL-4-APC mAb (all from BD Biosciences, USA).

### Enzyme-linked immunosorbent assay

The expression levels of IFN-γ and IL-4 in mouse serum were measured 14 days after the final immunization using commercial ELISA kits (R&D Systems), following the manufacturer’s instructions. To determine the titers of antigen-specific IgG and its subtypes (IgG1, IgG2a) in serum and IgA in feces, synthetic ASFV antigen peptides (Sangon, China; listed in Table S4) were used. A 96-well plate was coated overnight with 5 μg/mL of the synthetic ASF protein peptides in carbonate coating buffer. Plates were then blocked with 2% BSA for 2 hours at 37°C. Mouse serum and fecal supernatants were added and incubated for 2 hours at 37°C, followed by three washes with PBST. For serum samples, 100 μL of HRP-conjugated goat anti-mouse IgG, IgG1, or IgG2a (Beyotime, 1:4000 dilution) was added, while for fecal supernatants, HRP-conjugated goat anti-mouse IgA (Beyotime, 1:4,000 dilution) was used. Plates were incubated for 1 hour at 37°C, washed three times with PBST, and then 100 μL of TMB substrate was added per well and incubated at 37°C for 10 minutes. The reaction was terminated by adding 2M H₂SO₄, and the absorbance was measured at 450 nm using a BioTek 800 TS Absorbance Reader with Gen5 software (USA).

### Quantitative real-time PCR

The spleen and MLN were collected from mice 14 days after the third immunization to assess the transcription levels of IL-4, IL-2, and IFN-γ. Total RNA was extracted using an RNA extraction kit (Beijing TransGen Biotech Co., Ltd., TransGen) and subsequently reverse-transcribed into cDNA using the PrimeScript RT reagent kit (Takara). Real-time quantitative PCR (qPCR) was carried out with TB Green Premix Ex Taq II (Takara) on a real-time fluorescence quantitative PCR system (Germany). The specific primer sequences are listed in Table S5, with β-actin serving as the reference gene. Relative mRNA expression levels were calculated using the 2^−ΔΔCt^ method.

### Immunofluorescence

Paraffin-embedded sections of the mouse duodenum were prepared, and immunofluorescence staining was performed to assess the activation of B cells. The staining procedures followed the established laboratory protocol (40). The antibodies used included B220-FITC (200×) and IgA-PE (200×), both obtained from BD Biosciences.

### Statistical analysis

All experimental data were obtained from at least three independent experiments and are presented as mean ± SEM. Statistical analysis was performed using GraphPad Prism 10 software via one-way ANOVA. Differences were considered statistically significant at *P < 0.05, **P < 0.01, and ***P < 0.001; NS indicates no significant difference.

## SUPPLEMENTAL MATERIAL

Fig. S1 aem.00279-26-s0001.tif Detection of heterologous protein expression on *L. plantarum* surface by immunofluorescence microscopy.

Fig. S2 aem.00279-26-s0002.tif *In vivo* tracking of NC8/Δalr-EGFP following oral administration.

Fig. S3 aem.00279-26-s0003.tif Immunization-induced CD4^+^IFN-γ^+^ T cell responses in mediastinal lymph node and spleen.

Fig. S4 aem.00279-26-s0004.tif Immunization-induced CD4^+^IL-4^+^ T cell responses in mediastinal lymph node and spleen.

Fig. S5 aem.00279-26-s0005.tif Immunization-induced CD8^+^IFN-γ^+^ T cell responses in mediastinal lymph node and spleen.

Fig. S6 aem.00279-26-s0006.tif Mucosal IgA^+^ B cell induction in Peyer's patches and MLN following oral immunization.

Supplemental legends aem.00279-26-s0007.docx Descriptive legends for Fig. S1 to S6.

Supplemental tables aem.00279-26-s0008.docx Tables S1 to S5.

ASM does not own the copyrights to Supplemental Material that may be linked to, or accessed through, an article. The authors have granted ASM a non-exclusive, world-wide license to publish the Supplemental Material files. Please contact the corresponding author directly for reuse.
