# Supplementary figures and images for: Oral administration of Lactiplantibacillus plantarum displaying multiple ASFV antigen proteins on the surface induces systemic immune responses in mice
Source: Appl Environ Microbiol. 2026 Apr 24;92(5):e00279-26. doi: 10.1128/aem.00279-26 (PMC13188916; doi:10.1128/aem.00279-26)

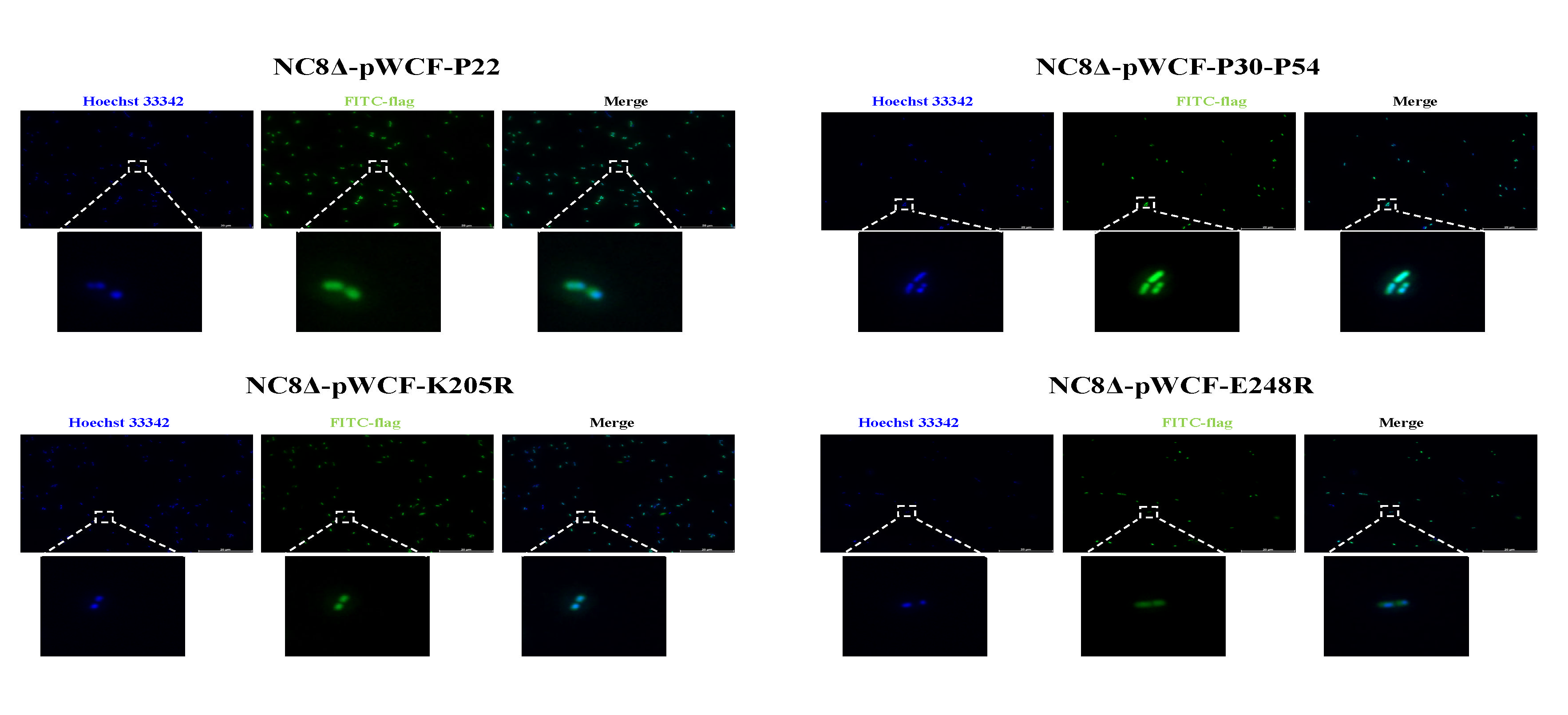

Supplement: Fig. S1 — Detection of heterologous protein expression on L. plantarum surface by immunofluorescence microscopy. [file aem.00279-26-s0001.tif]

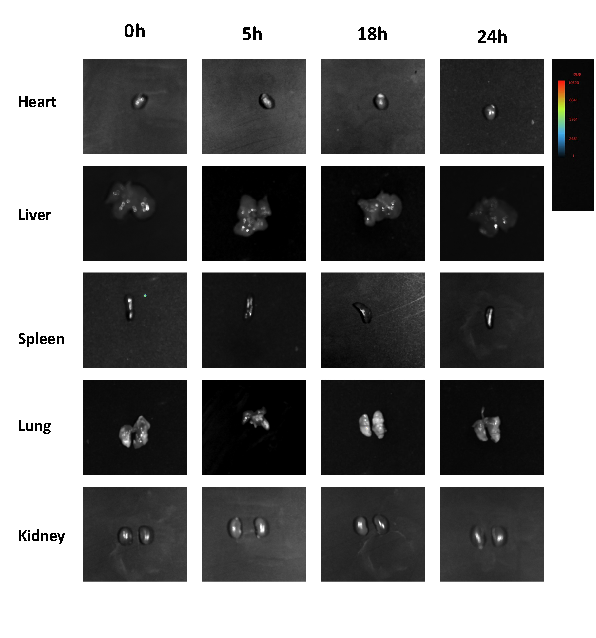

Supplement: Fig. S2 — In vivo tracking of NC8/Δalr-EGFP following oral administration. [file aem.00279-26-s0002.tif]

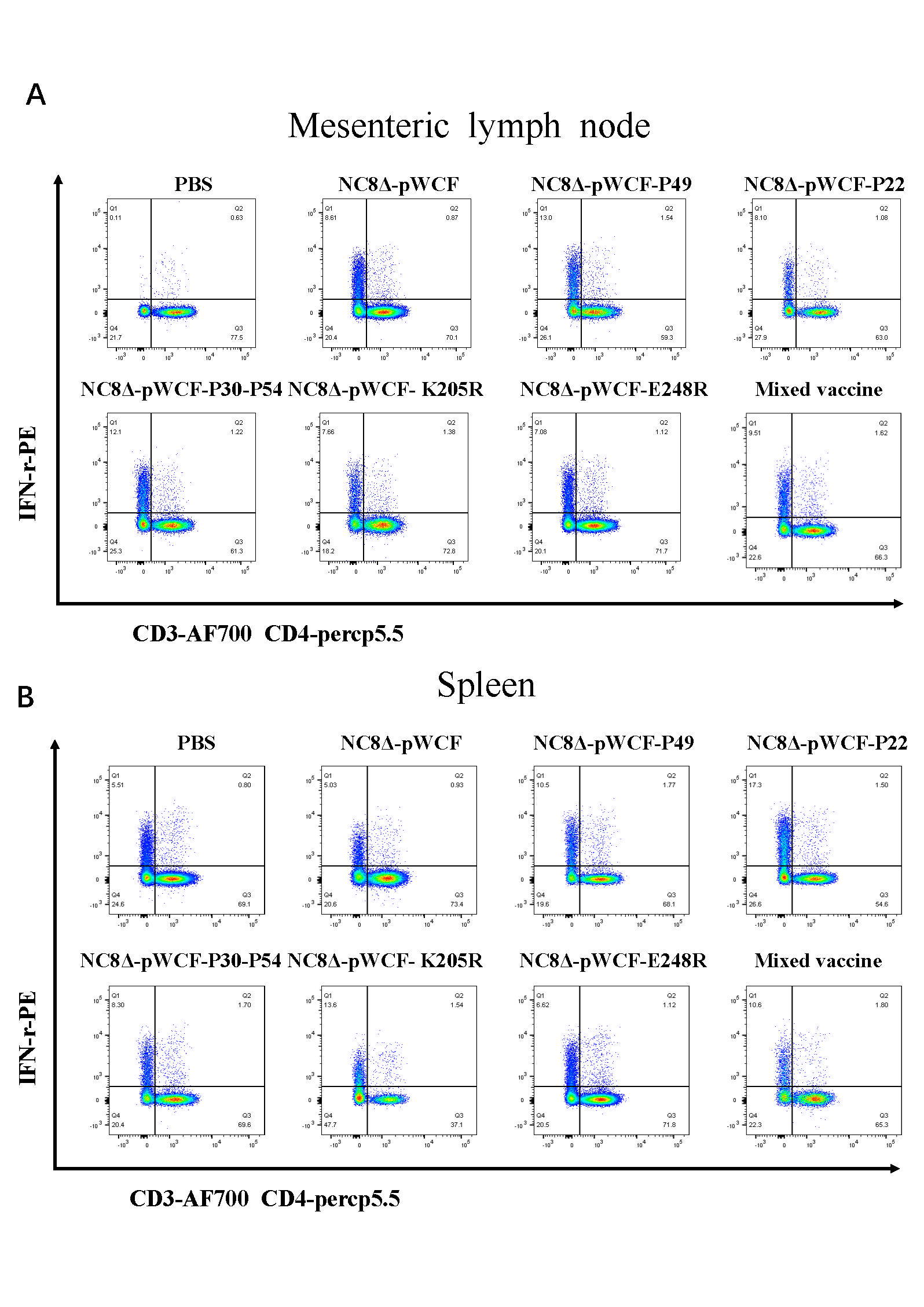

Supplement: Fig. S3 — Immunization-induced CD4+IFN-γ+ T cell responses in mediastinal lymph node and spleen. [file aem.00279-26-s0003.tif]

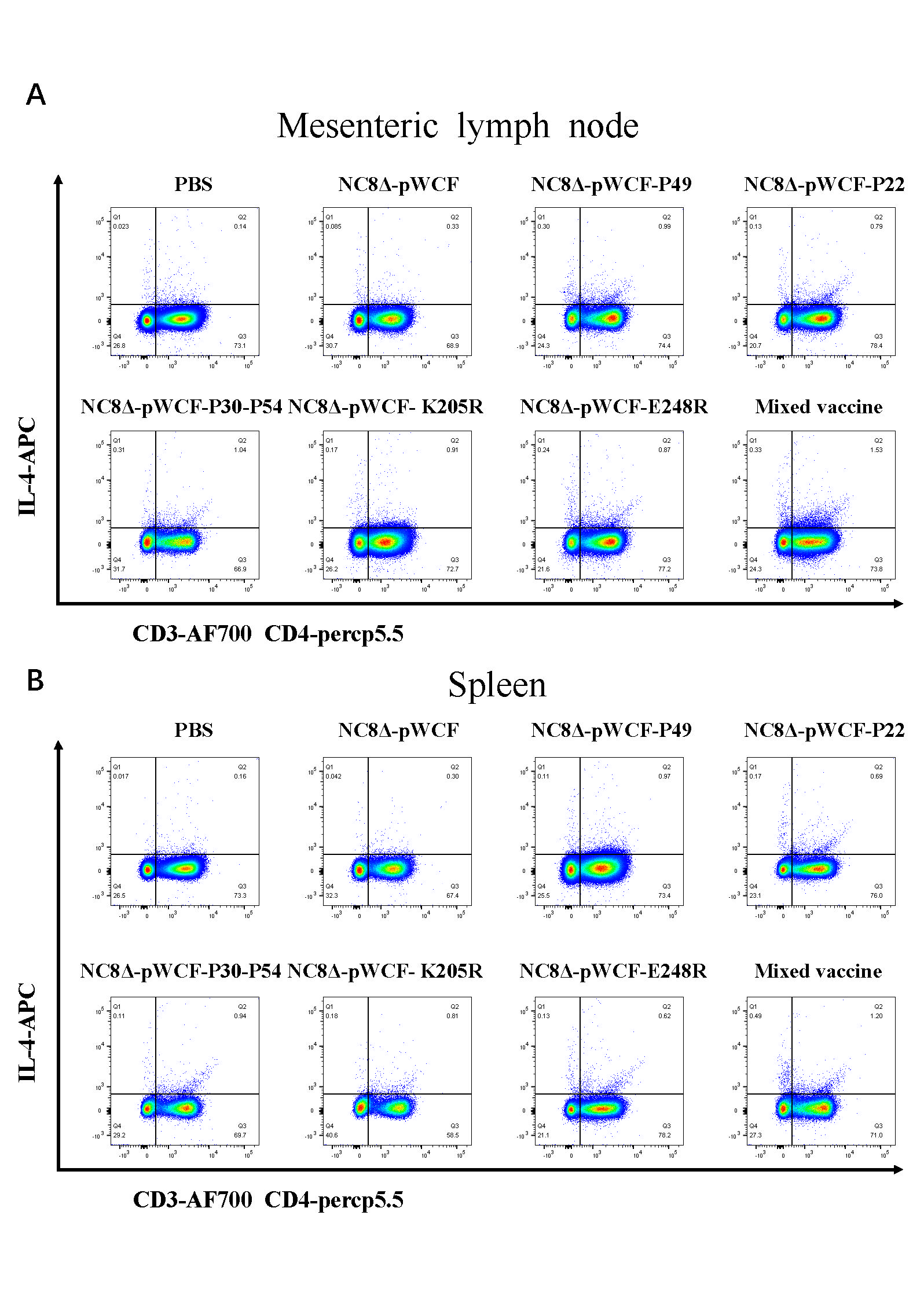

Supplement: Fig. S4 — Immunization-induced CD4+IL-4+ T cell responses in mediastinal lymph node and spleen. [file aem.00279-26-s0004.tif]

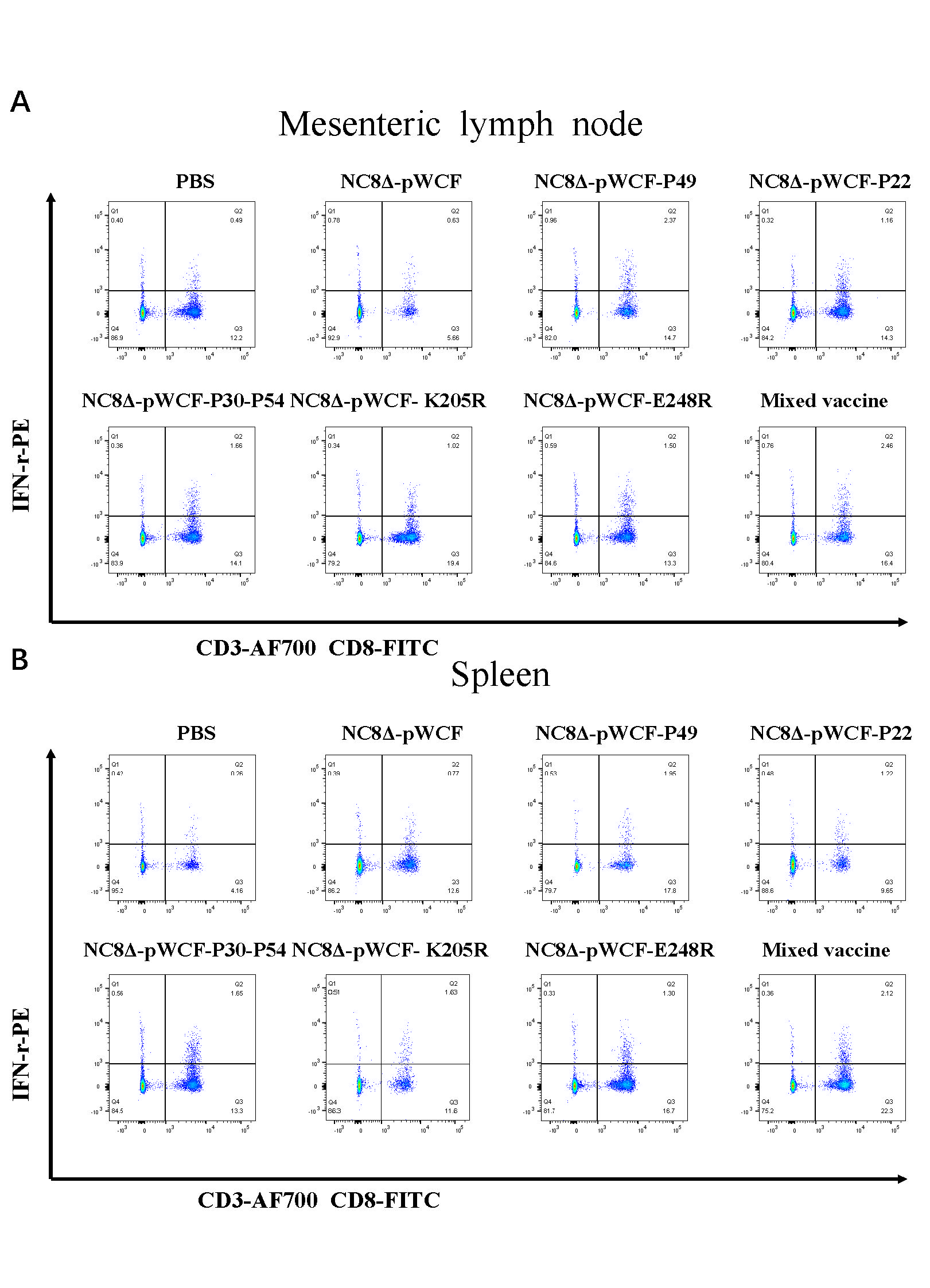

Supplement: Fig. S5 — Immunization-induced CD8+IFN-γ+ T cell responses in mediastinal lymph node and spleen. [file aem.00279-26-s0005.tif]

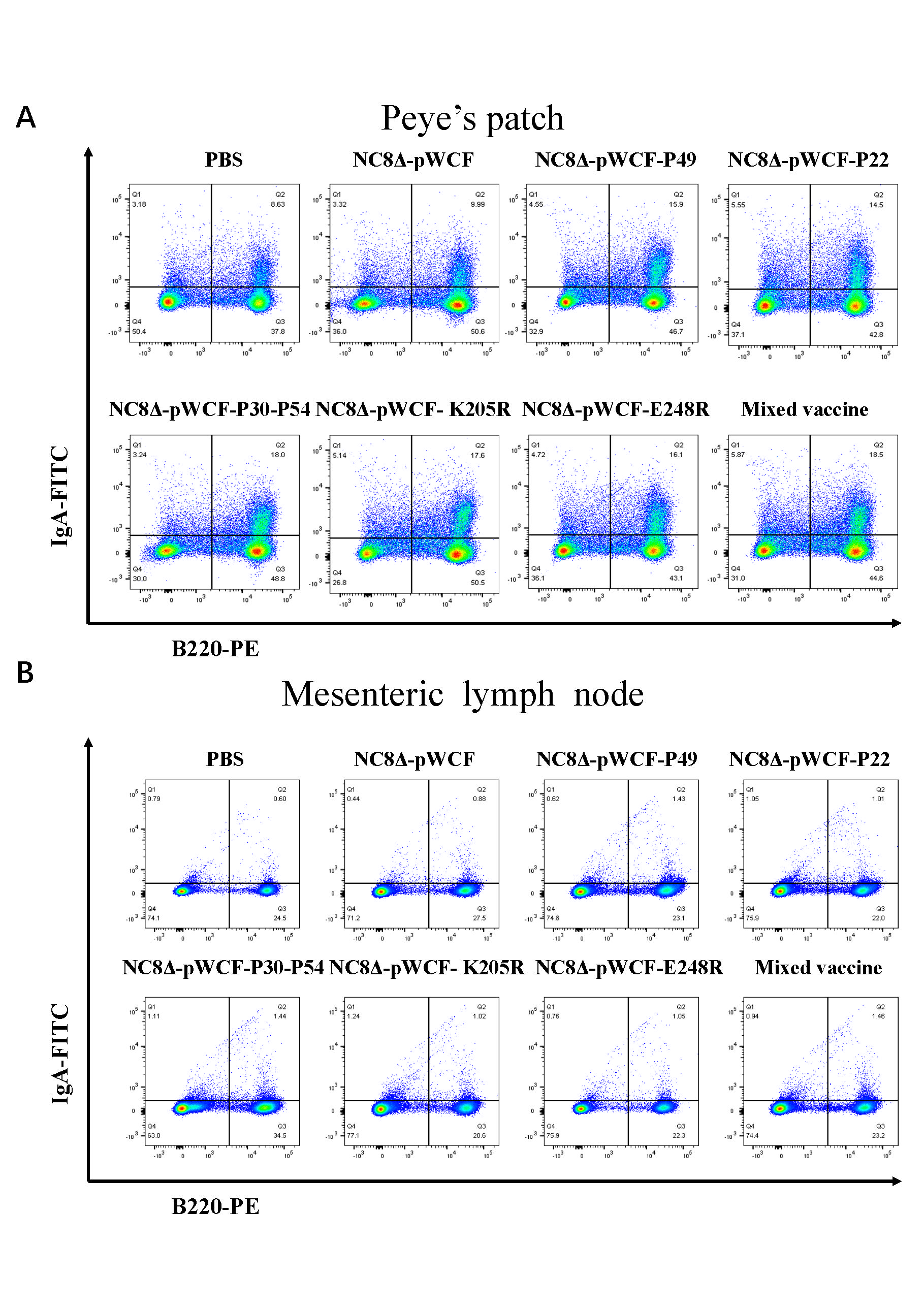

Supplement: Fig. S6 — Mucosal IgA+ B cell induction in Peyer's patches and MLN following oral immunization. [file aem.00279-26-s0006.tif]
