# Supplementary material for: Oral administration of Lactiplantibacillus plantarum displaying multiple ASFV antigen proteins on the surface induces systemic immune responses in mice
Source: Appl Environ Microbiol. 2026 Apr 24;92(5):e00279-26. doi: 10.1128/aem.00279-26 (PMC13188916; doi:10.1128/aem.00279-26)
Supplement: Supplemental legends — Descriptive legends for Fig. S1 to S6. [file aem.00279-26-s0007.docx]

**Supplementary FIGURE LEGENDs**

**Supplementary Fig. 1** **Detection of heterologous protein expression on *L. plantarum* surface by immunofluorescence microscopy.** Live bacteria were stained with Hoechst 33342 (blue) and incubated with FITC-conjugated mouse anti-FLAG monoclonal antibody to visualize heterologous protein expression on the bacterial surface (green). Merged images show co-localization. Scale bar, 20 μm.

**Supplementary Fig. 2 In vivo tracking of NC8/Δalr-EGFP following oral administration.** Following oral administration of engineered Lactobacillus plantarum NC8/Δalr (NC8-EGFP, 1×10⁹ CFU), no bacteria were detected in the heart, liver, spleen, or kidney of mice at designated time points. CFU enumeration confirmed exclusive gastrointestinal colonization without systemic dissemination (n=3 per group).

**Supplementary Fig. 3 Immunization-induced CD4⁺IFN-γ⁺ T cell responses in mediastinal lymph node and spleen.** Fourteen days after third immunization, the MLN and spleens of mice were collected, and frequency of CD4⁺IFN-γ⁺ T cells in the MLN (**A**) and spleen (**B**) were detected by flow cytometry (n = 5 mice per group).

**Supplementary Fig. 4 Immunization-induced CD4⁺IL-4⁺ T T cell responses in mediastinal lymph node and spleen.** Fourteen days after third immunization, the MLN and spleens of mice were collected, and frequency of CD4⁺IL-4⁺ T cells in the MLN (**A**) and spleen (**B**) were detected by flow cytometry (n = 5 mice per group).

**Supplementary Fig. 5 Immunization-induced CD8⁺IFN-γ⁺ T cell responses in mediastinal lymph node and spleen.** Fourteen days after third immunization, the MLN and spleens of mice were collected, and frequency of CD8⁺IFN-γ⁺ T cells in the MLN (**A**) and spleen (**B**) were detected by flow cytometry (n = 5 mice per group).

**Supplementary Fig. 6 Mucosal IgA⁺ B cell induction in Peyer's patches and MLN following oral immunization.** Five days after third immunization, PPs and MLN were collected from mice, and proportions of B220⁺IgA⁺ cells in the PP (**A**) and MLN (**B**) were detected by flow cytometry (n = 5 mice per group).
