# Supplementary material for: Oral administration of Lactiplantibacillus plantarum displaying multiple ASFV antigen proteins on the surface induces systemic immune responses in mice
Source: Appl Environ Microbiol. 2026 Apr 24;92(5):e00279-26. doi: 10.1128/aem.00279-26 (PMC13188916; doi:10.1128/aem.00279-26)
Supplement: Supplemental tables — Tables S1 to S5. [file aem.00279-26-s0008.docx]

**Table 1. Strains and plasmids used in this study.**

| **Strains** | **Brief Description** | **References/Sources** |
| --- | --- | --- |
| NC8/Δ*alr* | *Lactobacillus plantarum* NC8 *with alr gene deletion* | Lab stocks |
| *E. coli* χ6212 | *E.coli* host strain with asd mutation | Lab stocks |
| NC8Δ-pWCF | Expression of anchoring protein pgsA’ based on the 409ata vector | This study |
| NC8Δ-pWCF-P49 | Expression of fusion proteins of ASFV p49 with CTB anchored based on the 409ata vector | This study |
| NC8Δ-pWCF-P22 | Expression of fusion proteins of ASFV p22 with CTB anchored based on the 409ata vector | This study |
| NC8Δ-pWCF-P30-P54 | Expression of fusion proteins of ASFV p30 and p54 with CTB anchored based on the 409ata vector | This study |
| NC8Δ-pWCF-K205R | Expression of fusion proteins of ASFV pK205R with CTB anchored based on the 409ata vector | This study |
| NC8Δ-pWCF-E248R | Expression of fusion proteins of ASFV pE248R with CTB anchored based on the 409ata vector | This study |
|  |  |  |
| **Plasmids** | **Application** | **Source** |
| pSIP409-pgsA’ | *E. coli* -LAB shuttle plasmid, used for expressing target proteins in LAB, EryR. | Lab stocks |
| 409ata | *E. coli* -LAB shuttle plasmid, used for expressing target proteins in LAB, antibiotic-free. | Lab stocks |

**Table 2. Oligos used in this study.**

| **Primers** | **Sequence (5' → 3')** | **Application** |
| --- | --- | --- |
| 409ata-F | AATTCTATGAGTCGCTTTTTT | Construct an antibiotic-free ASFV plasmid |
| 409ata-R | CAACCAGCCGAATAATCCTTCTCGCTCACTGACTCGCTGC |  |
| M-F | agcgactcatagaattatttcctc |  |
| M-R | accgagcgcagcgagtcagtgagcgagaaggattat |  |

**Table 3**. Plasmid-encoded protein sequences

| **Code** | **Sequence** |
| --- | --- |
| NC8Δ-pWCF-P49 | **MGDYKDDDDKGDYKDDDDKIDYKDDDDKGSGGGGSGSKKELSFHEKLLKLTKQQKKKTNKHVFIAIPIVFVLMFAFMWAGKAETPKVKTYSDDVLSASFVGDIMMGRYVEKVTEQKGADSIFQYVEPIFRASDYVAGNFENPVTYQKNYKQADKEIHLQTNKESVKVLKDMNFTVLNSANNHAMDYGVQGMKDTLGEFAKQNLDIVGAGYSLSDAKKKISYQKVSRMYHDYASKLLADYRSDPPLWESDLPRHNRYSDNILNSRYCGNKNGAAPVYNEYTNSPEKAEKGLQLSDLRNFSFMLNPQHKNIGYGDAQDLEPYSSIPKNKLFNHFKNHRPAFSTHTENLIRRNVVRTEKKTFPQVASLKGTQKNCLTQPSSLPSLKNPKNSSVPSTRFSEHTKFFSYEDLPKLRTKGTIKHEQHLGDQMPGQHYNGYIPHKDVYNILCLAHNLPASVEKGIAGRGIPLGNPHVKPNIEQELIKSTSTYTDVPMLGPLPPKDSQHGREYQEFSANRHMLQVSNILHSVFANHSIKPQILEDIPVLNAQLTSIKPVSPFLNKAYQTHYMENIVTLVPRFKSIANYSSPIPNYSKRNSGQAEYFDTSKQTISRHNNYIPKYTGGIGDSKLDSTFPKDFNASSVPLTSAEKDHSLRGDNSACCISSISPSLGTIAAMIKLKFGVFFTVLLSSAYANGTPQNITDLCAEYHNTQIHTLNDKIFSYTESLAGKREMAIITFKNGATFQVEVPGSQHIDSQKKAIERMKDTLRIAYLTEAKVEKLCVWNNKTPHAIAAISMANHHHHHH** |
| NC8Δ-pWCF-P22 | **MGDYKDDDDKGDYKDDDDKIDYKDDDDKGSGGGGSGSKKELSFHEKLLKLTKQQKKKTNKHVFIAIPIVFVLMFAFMWAGKAETPKVKTYSDDVLSASFVGDIMMGRYVEKVTEQKGADSIFQYVEPIFRASDYVAGNFENPVTYQKNYKQADKEIHLQTNKESVKVLKDMNFTVLNSANNHAMDYGVQGMKDTLGEFAKQNLDIVGAGYSLSDAKKKISYQKVSRMRSSKKINNKKNMFNIKMTISTLLIALIILLIIILVVFLYYKKQQPPKKVCKVDKDCGSGEHCVRGSCSSLSCLDAVKMDKRNIKIDSKISSCEFTPNFYRFTDTAADEQQEFGKTRHPIKITPSPSESHSPQEVCEKYCSWGTDDCTGWEYVGDEKEGTCYVYNNPHHPVLKYGKDHIIALPRNHKHAGTIAAMIKLKFGVFFTVLLSSAYANGTPQNITDLCAEYHNTQIHTLNDKIFSYTESLAGKREMAIITFKNGATFQVEVPGSQHIDSQKKAIERMKDTLRIAYLTEAKVEKLCVWNNKTPHAIAAISMANHHHHHH** |
| NC8Δ-pWCF-K205R | **MGDYKDDDDKGDYKDDDDKIDYKDDDDKGSGGGGSGSKKELSFHEKLLKLTKQQKKKTNKHVFIAIPIVFVLMFAFMWAGKAETPKVKTYSDDVLSASFVGDIMMGRYVEKVTEQKGADSIFQYVEPIFRASDYVAGNFENPVTYQKNYKQADKEIHLQTNKESVKVLKDMNFTVLNSANNHAMDYGVQGMKDTLGEFAKQNLDIVGAGYSLSDAKKKISYQKVSRMVEPREQFFQDLLSAVDQQMDTVKNDIKDIMKEKTSFMVSFENFIERYDTMEKNIQDLQNKYEEMAANLMTVMTDTKIQLGAIIAQLEILMINGTPLPAKKTTIKEAMPLPSSNTNNEQTSPPASGKTSETPKKNPTNAMFFTRSEWASSNTFREKFLTPEIQAILDEQFANKTGIERLHAEGLYMWRTQFSDEQKKMVKEMMKKGTIAAMIKLKFGVFFTVLLSSAYANGTPQNITDLCAEYHNTQIHTLNDKIFSYTESLAGKREMAIITFKNGATFQVEVPGSQHIDSQKKAIERMKDTLRIAYLTEAKVEKLCVWNNKTPHAIAAISMANHHHHHH** |
| NC8Δ-pWCF-P30-P54 | **MGDYKDDDDKGDYKDDDDKIDYKDDDDKGSGGGGSGSKKELSFHEKLLKLTKQQKKKTNKHVFIAIPIVFVLMFAFMWAGKAETPKVKTYSDDVLSASFVGDIMMGRYVEKVTEQKGADSIFQYVEPIFRASDYVAGNFENPVTYQKNYKQADKEIHLQTNKESVKVLKDMNFTVLNSANNHAMDYGVQGMKDTLGEFAKQNLDIVGAGYSLSDAKKKISYQKVSRMDSEFFQPVYPRHYGECLSPVTTPNFFSTHMYTILIAIVVLVIIIIVLIYLFSSRKKKAAAIEEEDIQFINPYQDQQWVEVTPQPGTSKPAGATTASVGKPVTGRPATNRPATNKPVTDNPVTDRLVMATGGPAAAPAAASAPAHPAEPYTTVTTQNTASQTMSAIENLRQRNTYTHKDLENSLAEAAAKEAAAKEAAAKEAAAKALEAEAAAKEAAAKEAAAKEAAAKAMDFILNISMKMEVIFKTDLRSSSQVVFHAGSLYNWFSVEIINSGRIVTTAIKTLLSTVKYDIVKSARIYAGQGYTEHQAQEEWNMILHVLFEEETESSASSENIHEKNDNETNECTSSFETLFEQEPSSEVPKDSKLYMLAQKTVQHIEQYGKAPDFNKVIRAHNFIQTIYGTPLKEEEKEVVRLMVIKLLKKINFFLTYIGTIAAMIKLKFGVFFTVLLSSAYANGTPQNITDLCAEYHNTQIHTLNDKIFSYTESLAGKREMAIITFKNGATFQVEVPGSQHIDSQKKAIERMKDTLRIAYLTEAKVEKLCVWNNKTPHAIAAISMANHHHHHH** |
| NC8Δ-pWCF-E248R | **MGDYKDDDDKGDYKDDDDKIDYKDDDDKGSGGGGSGSKKELSFHEKLLKLTKQQKKKTNKHVFIAIPIVFVLMFAFMWAGKAETPKVKTYSDDVLSASFVGDIMMGRYVEKVTEQKGADSIFQYVEPIFRASDYVAGNFENPVTYQKNYKQADKEIHLQTNKESVKVLKDMNFTVLNSANNHAMDYGVQGMKDTLGEFAKQNLDIVGAGYSLSDAKKKISYQKVSRMGGSTSKNSFKNTTNIISNSIFNQMQSCISMLDGKNYIGVFGDGNILNHVFQDLNLSLNTSCVQKHVNEENFITNLSNQITQNLKDQEVALTQWMDAGTHDQKTDIEENIKVNLTTTLIQNCVSSLSGMNVLVVKGNGNIVENATQKQSQQIISNCLQGSKQAIDTTTGITNTVNQYSHYTSKNFFDFIADAISAVFKNIMVAAVVIVLIIVGFIAVFYFLHSRHRHEEEEEAEPLISNKVLKNAAVSGTIAAMIKLKFGVFFTVLLSSAYANGTPQNITDLCAEYHNTQIHTLNDKIFSYTESLAGKREMAIITFKNGATFQVEVPGSQHIDSQKKAIERMKDTLRIAYLTEAKVEKLCVWNNKTPHAIAAISMANHHHHHH** |

*Sequences highlighted in red represent pgsa′; blue indicates theASFV antigen protein; purple indicates CTB; black indicates linkers; and orange indicates the Flag tag.

**Table 4 Epitopes sequences used in Elisa experiments**

| **Antigen** | **Epitope sequence** |
| --- | --- |
| pE248R | NCVSSLSGMNVLVVKG |
| p49 | HKDVYNILCLAHNLPASVEK |
| p30 | ETNECTSSFET |
| p54 | YTHKDLENSL |
| p22 | SWGTDDCTGWEYVGDEKEGTC |
| pK205R | PTNAMFFTRSEW |

**Table 5 Specific primer sequences used in qPCR experiments**

| **Gene** | **Forward Sequence (5′–3′)** | | **Reverse Sequence (5′–3′)** |
| --- | --- | --- | --- |
| IFN-γ | | CTTGAAAGACAATCAGGCCATC | CTTGGCAATACTCATGAATGCA |
| IL-4 | | TGAATGACTGGAGGAGCTGAGACC | CGGAGTGGCGACATCGTACATAAC |
| IL-2 | | TGAGCAGGATGGAGAATTACAG | CAGAGGTCCAAGTTCATCTTCT |
| ACT-β | | CTACCTCATGAAGATCCTGACC | CACAGCTTCTCTTTGATGTCAC |
